# The histone demethylase JMJD2A/KDM4A links ribosomal RNA transcription to nutrients and growth factors availability

**DOI:** 10.1038/ncomms10174

**Published:** 2016-01-05

**Authors:** Kader Salifou, Swagat Ray, Laure Verrier, Marion Aguirrebengoa, Didier Trouche, Konstantin I. Panov, Marie Vandromme

**Affiliations:** 1 Centre for Integrative Biology, Université de Toulouse, UT3 Toulouse, France; 2 Centre for Integrative Biology, CNRS, UT3 Toulouse, France; 3 School of Biological Sciences, Queen's University Belfast, Belfast BT9 7BL, UK; 4 Centre for Cancer Research and Cell Biology, Queen's University Belfast, Belfast BT9 7AE, UK

## Abstract

The interplay between methylation and demethylation of histone lysine residues is an essential component of gene expression regulation and there is considerable interest in elucidating the roles of proteins involved. Here we report that histone demethylase KDM4A/JMJD2A, which is involved in the regulation of cell proliferation and is overexpressed in some cancers, interacts with RNA Polymerase I, associates with active ribosomal RNA genes and is required for serum-induced activation of rDNA transcription. We propose that KDM4A controls the initial stages of transition from ‘poised', non-transcribed rDNA chromatin into its active form. We show that PI3K, a major signalling transducer central for cell proliferation and survival, controls cellular localization of KDM4A and consequently its association with ribosomal DNA through the SGK1 downstream kinase. We propose that the interplay between PI3K/SGK1 signalling cascade and KDM4A constitutes a mechanism by which cells adapt ribosome biogenesis level to the availability of growth factors and nutrients.

## Abstract (web summary)

Histone methylation regulates gene expression. Here, Salifou *et al.* show that KDM4A, a histone lysine demethylase, regulates RNA polymerase I-driven transcription in a PI3K dependent manner, therefore linking nutrients and growth factors availability to ribosomal RNA transcription.

## Graphical abstract

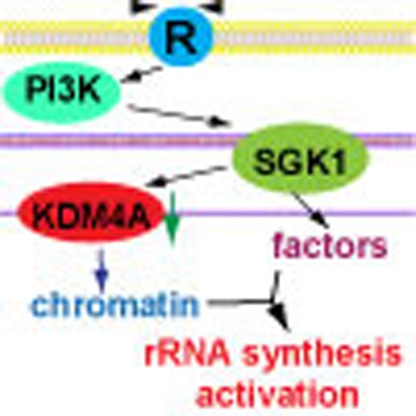

Eukaryotic genomic DNA, in a complex with histone proteins, is organized into a densely packed DNA–protein complex called chromatin, which harbours different levels of structural complexity and hierarchy^1^. Chromatin structure regulates DNA accessibility and therefore modulates the activity of enzymatic complexes requiring access to DNA. These complexes are involved in major cellular processes such as transcription, replication and DNA repair. Chromatin architecture is dynamic and regulated by DNA methylation^2^, chromatin remodelers^3^ and various histone modifications^4^. These epigenetic modifications play crucial roles in determining cell fate and the cellular response to external and internal stimuli.

The basic unit of chromatin is the nucleosome composed of 146 bp of DNA wrapped around an octamer of histones (two copies of each histone H2A, H2B, H3 and H4). Post-translational modifications of histones such as acetylation, phosphorylation or methylation are central in the regulation of chromatin structure. Histone modifications are reversible through the action of enzymes carrying antagonist activities. One of the key components of epigenetic regulation of transcription is the balance between methylation and demethylation of lysine residues in histones. Enzymes methylating lysines (lysine methyl transferases, KMTs) and enzymes removing methyl groups from lysines (histone demethylases, KDMs) are highly specific for given lysine residues. Many lysine residues in histones H3 and H4 can be mono- (me), di- (me2) or trimethylated (me3), including lysine 9 (K9), lysine 36 (K36) and lysine 4 (K4) on H3. H3K9 methylation is enriched in heterochromatin and is associated with the promoters of repressed genes in euchromatin. By contrast, methylation of H3K4 at the promoter, or H3K36 in the coding region mark active genes in euchromatin.

Ribosomal DNA (rDNA) encodes the 47S precursor of the 28S, 18S and 5S ribosomal RNA (rRNA) that are the main RNA components of ribosomes. Transcription of rRNA genes by RNA Polymerase I (Pol-I) is a key stage of ribosome biogenesis is directly linked to cell growth and proliferation and is regulated by a variety of signalling cascades including PI3K, mTOR and MAPK pathways^5^,^6^. Eukaryotic genomes contain a large number of rDNA repeats (in humans ∼350 copies) described to exist in three distinct chromatin states: epigenetically silenced heterochromatin which is maintained throughout the life of a cell, and two different forms of transcriptionally competent euchromatin: non-transcribed, ‘closed' chromatin and actively transcribed ‘open' chromatin^7^,^8^. The currently accepted model of rDNA transcription regulation in higher eukaryotes suggests that the number of epigenetically silenced rDNA genes is maintained during a normal cell cycle, but it can be modified during development, differentiation and disease^9^,^10^. The euchromatic rDNA copies are those subjected to transcriptional regulation in response to routine variations in external conditions (for example, nutrients, growth factors, stresses), to link rRNA synthesis to environmental conditions. The efficiency of rRNA synthesis at these ‘euchromatic' copies is regulated by a combination of two non-exclusive mechanisms: through the alteration of the rate of transcription and of Pol-I density and through epigenetic mechanisms that allow the passage from the closed to open chromatin states, such as post-translational modifications of histones and the re-positioning of nucleosomes^8^,^11^,^12^. However, how chromatin architecture is controlled by growth factors/nutrients is still poorly understood despite continued efforts.

In this manuscript, we report the involvement of the histone demethylase KDM4A in the regulation of rDNA transcription. As a member of the KDM4 family, KDM4A (also called JMJD2A or JHDM3A) is specific for H3K9me2/3 and H3K36me2/3 (refs 13, 14). Previous studies showed that KDM4A influences cell cycle progression and cell proliferation either through transcriptional control of key regulators of the cell cycle or by regulating replication timing^15^,^16^. Interestingly, it was recently shown that KDM4A also regulates protein translation in the cytosol^17^.

Here, we describe that KDM4A localizes within nucleoli, interacts with Pol-I machinery, binds to rDNA promoter and favours serum-stimulated rDNA transcription by demethylating H3K9me3. Moreover, we show that KDM4A nucleolar localization, and therefore its recruitment to chromatin, is controlled by the PI3K–SGK1 pathway, which is activated by growth factor/nutrients. Thus, our results demonstrate KDM4A as a growth factor/nutrients-dependent regulator of rDNA transcription, and highlight the key role of KDM4A in the processes that regulate cell growth and proliferation.

## Results

### KDM4A is a nucleolar protein and associates with rDNA

**Figure 1:**
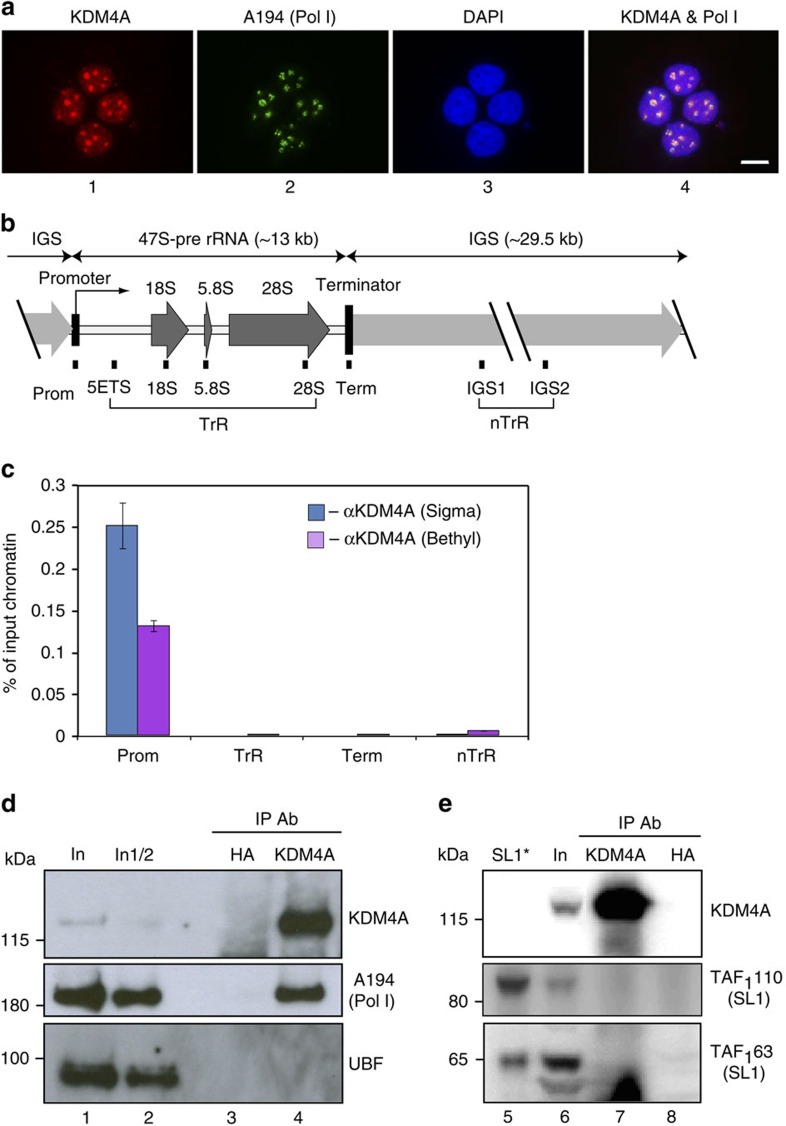
KDM4A is a nucleolar protein. (**a**) Actively growing U2OS cells (confluency 60–70%) were fixed following extraction with 1% Triton X-100 and analysed by indirect immunofluorescence using antibodies specific to human KDM4A (Ab1 Bethyl Lab, panel 1) and human Pol-I largest subunit A194 (panel 2); nuclear DNA was stained by DAPI (panel 3). Images were acquired with a Leica fluorescent microscope (DM5000- 20 × objective). Representative nuclei are shown with associated merged images (panel 4). Scale bar, 5 μM. (**b**) A diagram of the human rDNA repeat is shown indicating the positions of eight sets of specific PCR primer/probes used for qPCR analysis of immunoprecipitated DNA. 5′ETS, 5′-external transcribed spacer; IGS, intergenic spacer; Prom, the rRNA promoter, term, the terminator. Signal representing the transcribed region (TrR) is the average of the combined signal from 5′ETS, 18S, 5.8S and 28S rRNA. Signal representing the non-transcribed region (nTrR) is the average of the combined signal from IGS1 and IGS2. (**c**) ChIP assays were performed using antibodies specific to human KDM4A from two different sources and analysed by qPCR using eight sets of specific probes and primers derived from different regions of rDNA repeats (see the diagram above). The value of each bar represents the difference between the signals from the specific antibody and from the negative control (an appropriate IgG) expressed as % from total chromatin input (see Supplementary Fig. 3a for raw data). Signal representing the transcribed region (TrR) is the average of the combined signal from 5′ETS, 18S, 5.8S and 28S rRNA. Signal representing the non-transcribed region (nTrR) is the average of the combined signal from IGS1 and IGS2. The s.d.'s from three independent experiments are shown; *n*=3. (**d**, **e**) KDM4A was immunoprecipitated from nuclear extract of U2OS cells using KMD4A-specific antibodies (Bethyl). Immunoprecipitated complexes were analysed by western blotting using either antibodies specific to human Pol-I largest subunit A194 and UBF (lane 4), or to SL1 subunits TAF_1_110 and TAF_1_63 (lane 7). Input—lanes 1, 2 and 6, negative control (anti-HA antibodies)—lanes 3 and 8. Purified SL1—lane 5. Positions of prestained molecular weight markers (PageRuler Plus, Fermentas) are indicated.

KDM4A subcellular localization was determined in growing U2OS cells by indirect immunofluorescence using an antibody directed against the C-terminal part of KDM4A (anti-KDM4A-Ab1, Fig. 1a). As expected, KDM4A was found in the nucleus. Interestingly, inside the nucleus, the staining was not uniform and we observed that darker zones of 4′,6′-diamindino-2-phenylindole (DAPI) stained nuclear DNA, which correspond to nucleoli, exhibited a brighter staining. Moreover, KDM4A co-localized with the largest subunit of Pol-I (A194, Fig. 1a) and other nucleolar proteins (fibrillarin or UBF, a Pol-I transcription factor (Supplementary Fig. 1a)). Importantly, a similar pattern was also observed using a second antibody directed against the N-terminal part of KDM4A (anti KDMA-Ab2, Supplementary Fig. 1b), indicating that the signal is indeed specific to KDM4A and not due to any cross-reaction with another protein. Note that this nucleolar localization was observed both by classical immunofluorescence (Supplementary Fig. 1b), or following extraction of soluble proteins by triton treatment before fixation (Fig. 1a and Supplementary Fig. 1a), indicating that KDM4A is tightly bound inside the nucleolus.

Similar results were observed in other cells, such as transformed cell lines (HeLa, GC92) or primary fibroblasts (WI38; Supplementary Fig. 2a). Noticeably, by contrast to KDM4A, the histone demethylase LSD1 was excluded from the nucleolus of U2OS cells (Supplementary Fig. 2b), indicating that nucleoli accumulation is not a general feature of KDMs.

Nucleoli are the sites of rDNA transcription driven by RNA Pol-I. rDNA genes, which encode the 47S rRNA precursor are present in multiple copies in the human genome (>300 copies) and localize inside the nucleoli during interphase. Considering that KDM4A is a chromatin modifier, we next tested its association with rDNA. We performed chromatin immunoprecipitation (ChIP) experiments using chromatin from growing U2OS cells and analysed KDM4A occupancy at various regions from the rDNA repeats by quantitative PCR (qPCR; Fig. 1b,c). To present specific changes at transcribed (TrR) and non-transcribed (nTrR) regions of rDNA, we averaged signals from four amplicons in TrR and from two in nTrR (Fig. 1b). Values obtained using the different probes along the transcribed (or non-transcribed) region were generally very similar (see for example Supplementary Fig. 9).

We observed a strong enrichment of rDNA promoter regions in ChIPs performed using two different KDM4A-specific antibodies, and a low enrichment of other rDNA regions (transcribed, terminator and intergenic regions, (Fig. 1c, see Supplementary Fig. 3a for raw data), indicating that KDM4A is specifically associated with the rDNA promoter in growing U2OS cells.

This specific association led us to test whether KDM4A can interact with the Pol-I transcription machinery. We immunoprecipitated endogenous KDM4A from U2OS nuclear extracts and analysed immunoprecipitated complex by Western blotting using antibodies specific to the large subunit of Pol-I A194, Pol-I specific transcription factor UBF and two subunits of Pol-I promoter recognition factor SL1 (TAF_I_110 and TAF_I_63). We found that Pol-I, but not UBF or SL1, co-precipitated with KDM4A, demonstrating that endogenous KDM4A and Pol-I are present within the same multimolecular complex (Fig. 1d).

Altogether, these results show that KDM4A is a nucleolar protein that interacts with Pol-I and associates with the rDNA promoter, suggesting that it may play a role in regulating rDNA transcription.

### KDM4A is dispensable for steady-state rRNA synthesis

**Figure 2:**
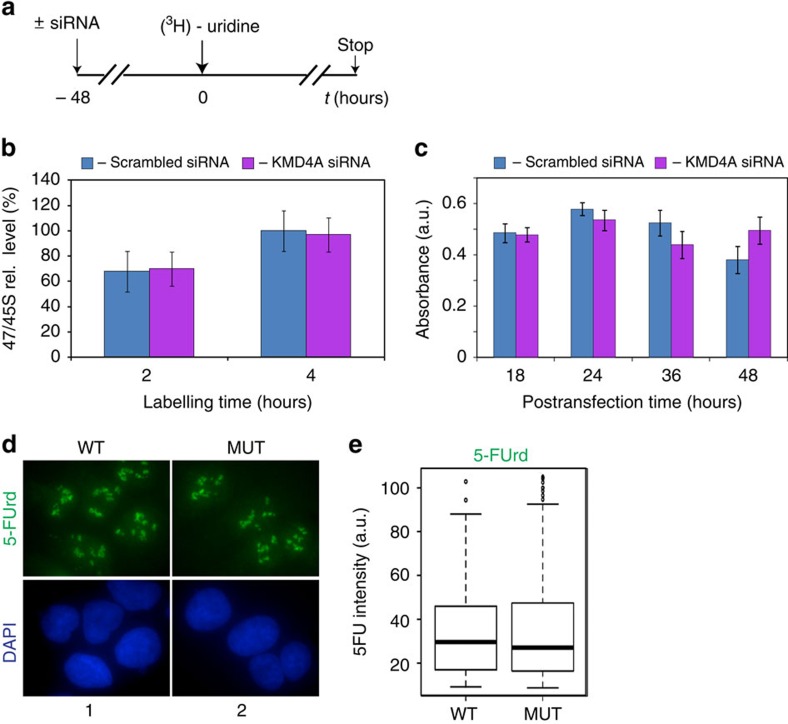
KDM4A is not essential for ongoing Pol-I transcription. (**a**) Schematic representation of the labelling of cells with ^3^H-uridine to determine the effect of KDM4A depletion on ongoing rRNA synthesis. RNA was extracted 2 and 4 h after ^3^H-uridine addition and *de nov*o rRNA transcripts were detected by tritium imaging of RNA blots as in Fig. 3b. (**b**) The relative efficiencies of rRNA synthesis were quantitated as in Fig. 3 and transcript levels are indicated for 47S/45S pre-rRNA. The data, expressed as a percentage of the highest value (set at 100%). The s.d.'s for three independent experiments are shown; *n*=3. (**c**) The relative efficiencies of cell growth were determined using MTT assay. The s.d.'s for four independent samples are shown; *n*=4. (**d**) Growing U2OS cells (∼70% confluent) were electroporated with an expression vector encoding either wild-type KDM4A (WT) or catalytically inactive mutant (MUT). Cells were grown for 48 h and then 5-FUrd was added for 30 min and cells were fixed and analysed by indirect immunofluorescence using antibodies specific to 5-FUrd (anti-BrdU antibodies); nuclear DNA was stained by DAPI. (**e**) Box-and-whiskers plot of the quantification of 5-FUrd staining per cell (*n*=100–200) obtained from the experiment shown in **d** using R open source software (a.u. stands for arbitrary units). Note that the 5-FUrd levels of the cell populations overexpressing the MUT and the WT are not significantly different (*P* value=0.637, Wilcoxon test). The median values are shown as horizontal lines, outliers are shown as open circles; *n*=3.

Because KDM4A is present at the rDNA promoter in growing cells (Fig. 1c), we analysed its involvement in Pol-I transcription in asynchronously growing cells. Neither siRNA-mediated knockdown of KDMA4 (Fig. 2a,b) nor overexpression of a demethylase defective mutant (Fig. 2d,e) (working as a dominant-negative mutant in starved–refed conditions, see below) had major effects on the level of rDNA transcription in growing U2OS cells (Fig. 2b,e) or on U2OS cells proliferation (Fig. 2c). Thus, although KDM4A is associated with rDNA promoter in growing cells suggesting a potential role for this enzyme, it does not seem to be important for efficient rDNA transcription. We cannot rule out the possibility that combination of low requirements for KDM4A in growing cell and <100% efficiency of siRNA-mediated depletion and endogenous KDM4A inhibition led to the apparent lack of the effects of KDM4A siRNA and dominant-negative mutant overexpression in growing cells. Nevertheless, these findings suggest that KDM4A is largely dispensable for the maintenance of rDNA repeats transcription.

### KDM4A is required for serum-induced rDNA transcription

rDNA transcription is tightly regulated by the supply in growth factors and nutrients that impact on the energy status of the cell and regulate intracellular metabolites levels^18^. Serum contains both growth factors and nutrients and, as a consequence, rRNA production is repressed upon serum deprivation and re-activated upon serum addition. This serum-dependent regulation of rDNA transcription is associated with chromatin remodelling and changes in histone modifications (for latest review see ref. 19).

**Figure 3:**
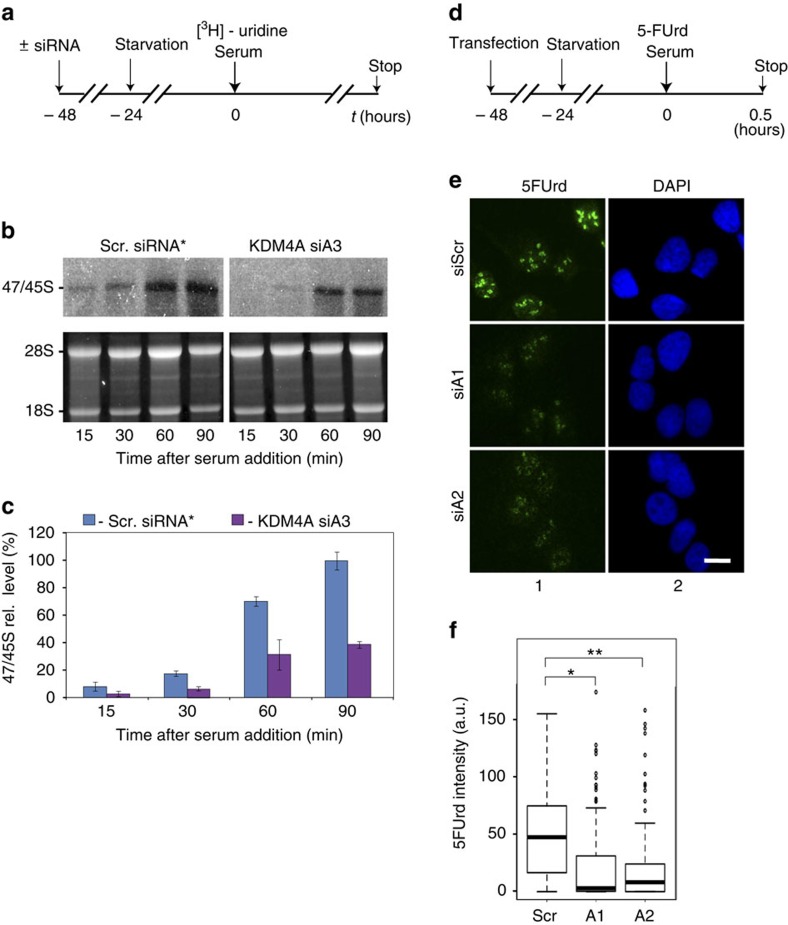
KDM4A is important for serum-induced rRNA synthesis. (**a**) Schematic representation of the labelling of cells with ^3^H-uridine to determine the effect of KDM4A depletion on activation of rRNA synthesis. U2OS cells were transfected either with non-targeting (siScr) or KDM4A-specific siRNA3 (siA3). 24 h post-transfection cells were starved. Transcription was activated by addition of serum and ^3^H-uridine at time point 0. (**b**) RNA was extracted 15, 30, 60 and 90 min after serum addition and *de nov*o rRNA transcripts were detected by tritium imaging of RNA blots (top panel) using X-ray film. Total 18S and 28S rRNAs were detected by ethidium bromide staining (bottom panel). (**c**) To determine the relative efficiencies of rRNA synthesis, RNA blots were imaged using tritium image plate (Fuji) and quantitated with aid of phosphoimager (Fuji) and Aida software (Raytec). Transcript levels are indicated for 47S/45S pre-rRNA. The data are expressed as a percentage of the highest value (set at 100%). The s.d.'s for three independent experiments are shown; *n*=3. (**d**). Schematic representation of the labelling of cells with 5-FUrd to determine the effect of KDM4A depletion on activation of rRNA synthesis in individual cells. U2OS cells were serum-starved for 16 h, electroporated with either a non-targeting siRNA (siScr) or two siRNAs directed against KDM4A (siA1 and siA2) and kept in serum-free medium for further 24 h before serum refeeding in the presence of 5-FUrd for 30 min. (**e**). Cells were stained for 5-FUrd incorporation using anti-BrdU antibodies. Nuclear DNA was stained with DAPI. Representative immunofluorescence images are shown. Scale bar, 5 μM. (**f**). Quantification of 5-FUrd staining shown in **e**, done as in Fig. 2e; *P* value (Wilcoxon test)=*4.84 × 10^−11^, **6.12 × 10^−12^. Note the presence of many outliers in samples transfected by KDM4A siRNA, which most likely correspond to untransfected cells. The median values are shown as horizontal lines, outliers are shown as open circles; *n*=3.

We thus tested the effect of KDM4A depletion on *de novo* rRNA synthesis in starved–refed cells. Serum-starved cells were transfected with a siRNA directed against KDM4A or a non-targeting siRNA as a control before stimulation by serum (see Supplementary Fig. 4 for siRNA efficiency) and rRNA expression was analysed by ^3^H-Uridine labelling at different times following serum addition (Fig. 3a). As expected, the expression of pre-RNA transcripts was activated following serum stimulation in control cells. However, this activation was significantly reduced in KDM4A-depleted cells (Fig. 3b,c), demonstrating that KDM4A is important for serum-induced activation of the pre-rRNA production. A similar result was also observed when rRNA expression was assessed in individual cells by metabolic labelling with 5-Fluorouridine (5-FUrd; Fig. 3d–f). Notably, three different siRNA were used in these experiments, ruling out any off-target effects of siRNAs. Importantly, we found that KDM4A depletion did not affect the expression of UBF and Pol-I (Supplementary Fig. 4b) suggesting a direct role of KDM4A in the regulation of rRNA synthesis.

All together, these results demonstrate that KDM4A is specifically involved in the activation of Pol-I-driven transcription in response to serum.

### KDM4A behaviour is regulated by serum

We next analysed KDM4A behaviour in starved and starved–refed cells. U2OS cells were serum starved and refed with serum as in Fig. 3 and RNA isolated from starved and refed cells were analysed by RT-qPCR (Supplementary Fig. 5a). As expected, the levels of the pre-rRNA increased following serum addition. However, no significant changes were observed in the mRNA levels of KDM4A, KDM4B or KDM4C histone demethylases. At the protein level, we observed a reproducible increase in KDM4A protein levels (by approximately twofold) upon serum addition, which was due to increased stability of KDM4A (Supplementary Fig. 5b,c).

**Figure 4:**
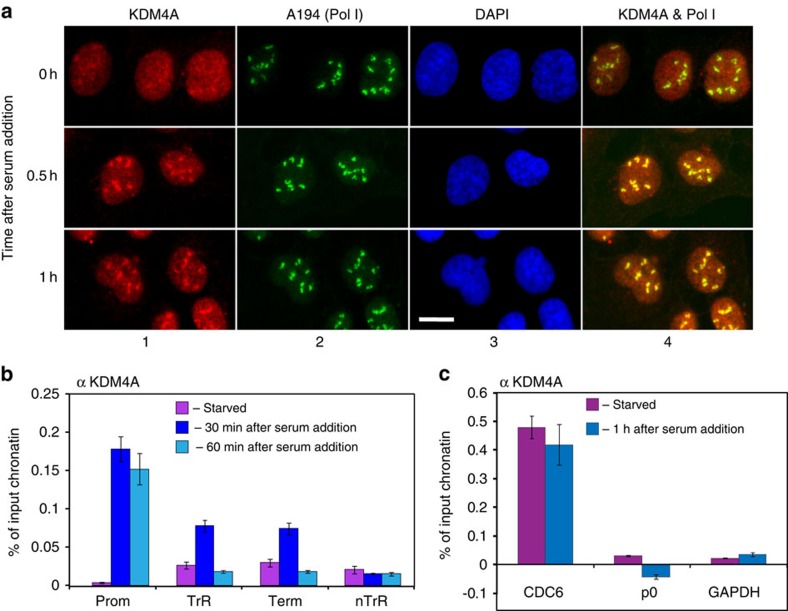
KDM4A localization and association with rDNA is controlled by serum. (**a**). U2OS cells were serum starved for 24 h (*t*=0) and refed with serum. Cells were fixed at 30 and 60 min following serum addition, and analysed by indirect immunofluorescence using antibodies specific to human KDM4A (Ab1 Bethyl Lab, panels 1) and human Pol-I largest subunit A194 (panels 2); nuclear DNA was stained by DAPI (panels 3). Merged images are shown (panel 4). Scale bar, 5 μM. (**b**) ChIP assays were performed using antibodies specific to human KDM4A (Ab1, Bethyl Lab) and chromatin isolated from starved and starved–refed cells (30 and 60 min after serum addition) and analysed as in Fig. 1c to determine KDM4A binding to rDNA promoter (Pr), transcribed region (TrR) or non-transcribed region (nTrR). The value of each bar represents the difference between the signals from the specific antibody and from the negative control (an appropriate IgG) expressed as % from total chromatin input (see Supplementary Fig. 3b for raw data). Standard deviations from three independent experiments are shown; *n*=3. (**c**) ChIP assays were performed using antibodies specific to human KDM4A as in **b** and chromatin immunoprecipitated from starved and starved–refed cells (60 min after serum addition) were analysed by qPCR for the presence either CDC6 or P0 or GAPDH promoters representing a positive and negative controls of the anti-KDM4A ChIP, respectively. The value of each bar represents the difference between the signals from the specific antibody and from the negative control (an appropriate IgG) expressed as % from total chromatin input (see Supplementary Fig. 3c for raw data). Standard deviations from three independent experiments are shown; *n*=3.

We also analysed KDM4A cellular localization in starved and starved–refed cells at two different time points after serum addition. We found that in starved cells, KDM4A largely lost its nucleolar localization, which was rapidly restored following serum addition (Fig. 4a; Supplementary Fig. 7a,b), indicating that KDM4A is relocalized to the nucleoli in response to serum. We next tested if KDM4A–rDNA association is also regulated by serum using ChIP approach. We found that in starved cells association of KDM4A with the rDNA promoter was nearly abolished (Fig. 4b; Supplementary Fig. 3b). However, it was restored as soon as 30 min after serum addition at a level that remained constant for 1 h. Importantly, such a serum-induced recruitment of KDM4A is specific to rDNA, since on a Pol-II-regulated target of KDM4A, the CDC6 promoter^20^, binding of KDM4A was unchanged upon serum stimulation (Fig. 4c; Supplementary Fig. 3c). Importantly, KDM4A association with DNA is limited to the specific *loci* and not detected at non-KDM4A dependent genes (Fig. 4c; Supplementary Fig. 3c). Thus, the increase of KDM4A occupancy at the rDNA is not a mere consequence of the global increase in KDM4A level but reflects the functional importance of the serum-dependent regulation of KDM4A nucleolar localization.

Interestingly, and by contrast to what we observed in growing cells (Fig. 1c), KDM4A was transiently recruited to the transcribed region of rDNA following serum stimulation, suggesting that KDM4A can have targets outside the promoter, perhaps allowing the first round of transcription. These results are consistent with our finding of a physical interaction between Pol-I and KDM4A (Fig. 1d), since Pol-I is present upon serum addition on both the rDNA promoter and the coding region^21^ (Supplementary Fig. 6a).

Taken together, these data indicate that the nucleolar localization of KDM4A and its association with rDNA are controlled by growth factor-/nutrient-dependent mechanisms.

### KDM4A favours rDNA transcription by demethylating H3K9

**Figure 5:**
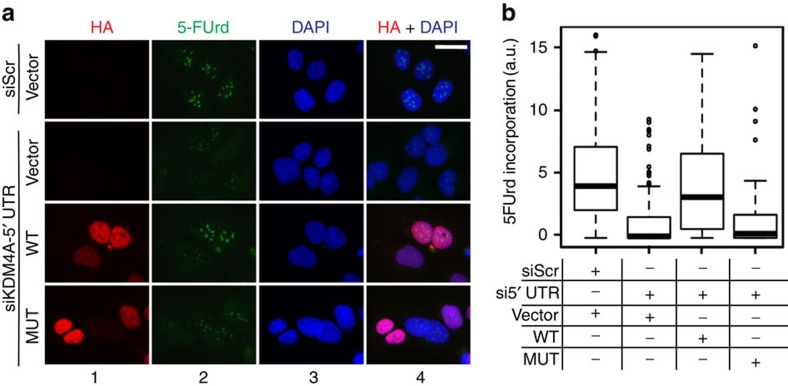
KDM4A activity is important for serum-induced rRNA synthesis. (**a**) U2OS cells were electroporated with an expression vector encoding either wild-type HA-tagged KDM4A (WT) or catalytically inactive mutant (MUT) or with empty vector (vector) together with a siRNA targeting the 5′UTR of KDM4A, to knockdown endogenous KDM4A while allowing expression of exogenous KDM4A proteins. Cells were grown for 24 h and then starved for another 24 h before serum refeeding in the presence of 5-FUrd. 30 min after serum addition cells were fixed and analysed by indirect immunofluorescence using antibodies specific to HA-tag to detect overexpressed proteins, (panels 1), 5-FUrd (panels 2); nuclear DNA was stained by DAPI (panels 3). Merged images are shown (panels 4). Bar=5 μM. (**b**) Quantification of 5-FUrd staining shown in **a**, done as in Fig. 2e. Note that the 5-FUrd levels of the cell populations overexpressing the MUT and the WT are significantly different (*P* value=4.97 × 10^−9^, Wilcoxon test). The median values are shown as horizontal lines, outliers are shown as open circles; *n*=3.

We next investigated the mechanism by which KDM4A participates in rRNA synthesis upon serum stimulation. We first tested whether KDM4A enzymatic activity is required for the regulation of Pol-I transcription by investigating the ability of wild-type KDM4A or a KDM4A mutant (KDM4AH188A) defective for histone demethylase activity^15^ to restore rDNA transcription in cells depleted from endogenous KDM4A. We transfected U2OS cells with siRNA targeting the 5′ untranslated region of KDM4A (so that it did not silence the expression of recombinant KDM4A which does not contain the 5′UTR), and we found, as expected, that this siRNA affect the efficiency of activation of rDNA transcription (Fig. 5a, column 2 and b). Strikingly, overexpression of wild-type KDM4A before starvation and refeeding, but not of the enzymatic-defective KDM4A, efficiently restored rDNA transcription (Fig. 5a,b; see Supplementary Fig. 8a for KDM4A expression). Moreover, in the absence of siRNA depletion of KDM4A, we observed that cells expressing inactive KDM4A exhibited decreased levels of rDNA transcription compared with wild type showing its dominant-negative effect (Supplementary Fig. 8b,c).

These results indicate that KDM4A demethylase activity is required for efficient activation of rRNA synthesis and that the inactive KDM4A functions as a dominant-negative mutant, suggesting the importance of changes in the methylation status of KDM4A substrates at rDNA genes.

The human genome contains >300 copies of rDNA repeats and the chromatin structure of these repeats is not uniform, rDNA genes being either hetero- or euchromatic^19^,^22^. Our ChIP data showed that KDM4A is associated with rDNA and this association is regulated by serum (Fig. 1c; Fig. 4b). However, these experiments do not allow to distinguish heterochromatic and euchromatic rDNA. Thus, we next investigated whether KDM4A associated with a particular subpopulation of rDNA genes. To this goal, we first isolated rDNA associated with Pol-I, corresponding to euchromatic rDNA, by immunoprecipitating chromatin with antibodies specific to the core Pol-I subunit (second largest subunit A135). Indeed, according to previous results^21^ and our data (Supplementary Fig. 6a), a significant fraction of Pol-I remains associated with rDNA promoter and transcribed regions in serum-starved cells which allows isolation of euchromatic rDNA.

This result stands in contrast to a previous report, which showed that Pol-I is mainly associated with the transcriptionally active pool of rDNA, and that its association is lost in starved cells^8^. In our hands, association of Pol-I with rDNA in various cell lines (U2OS, HeLa and MCF7) is only lost if the cells are under severe stress (that is, at least 48 h of serum deprivation) which is accompanied by changes in nucleolar morphology and by a significant decrease in cell viability (K.I.P., unpublished data). Therefore, the most likely explanation for this discrepancy is the different sensitivity of various cells to metabolic stresses and/or differences in starvation regime.

**Figure 6:**
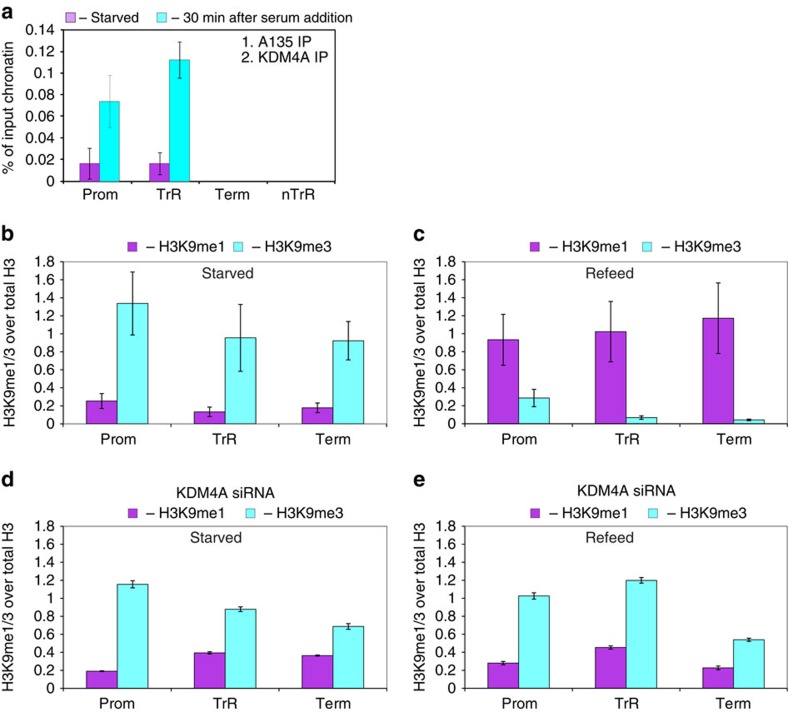
KDM4A is important for serum-induced histone demethylation at rDNA repeats. (**a**) Chromatin was isolated from starved and starved–refed cells (30 min after serum addition) and subjected for the first round of immunoprecipitation using antibody specific to Pol-I subunit A135. After elution chromatin was subjected to the second IP round using antibody specific to KDM4A and analysed by qPCR as in Fig. 1c. The value of each bar represents the difference between the signals from the specific antibody and from the negative control (an appropriate IgG) expressed as % from total chromatin input (see Supplementary Fig. 3d for raw data). Standard deviations from three independent experiments are shown; *n*=3. (**b**,**c**) Chromatin was isolated from starved (**b**) and starved–refed U2OS cells (1 h after serum addition) (**c**) and the same quantities of a chromatin were subjected for the first round of immunoprecipitation using antibody specific to Pol-I subunit A135. After elution, the chromatin was subjected for the second IP round using antibodies specific to histone H3, H3K9me1, H3K9me2 and H3K9me3 and analysed by qPCR as in Fig. 1c. The value of each bar represents the difference between the signals from the specific antibody and from the negative control (an appropriate IgG) normalized to the specific H3 signal (representing the difference between the signals from H3 specific antibody and an appropriate IgG, see Supplementary Fig. 6d for raw data) and expressed as % from total chromatin input. Standard deviations from three independent experiments are shown (see Supplementary Fig. 9a-c for raw data); *n*=3. (**d**,**e**). U2OS cells were transfected with KDM4A specific siRNA3. 24 h post-transfection cells were starved for 24 h. Chromatin was isolated from starved (**d**) and starved–refed cells (1 h after serum addition) (**e**). Chromatin was analysed as in **b** and **c** (see Supplementary Fig. 9d-f for raw data); *n*=3.

The Pol-I-associated chromatin was next subjected to a second round of immunoprecipitation using antibodies specific to KDM4A as well as non-modified or modified histones. Using this sequential ChIP (sChIP) approach we detected a significant signal of rDNA promoter regions following KDM4A sChIP, confirming that KDM4A associates with the euchromatic pool of rDNA repeats (Fig. 6a; Supplementary Fig. 3d).

We next investigated the dynamic of H3K9 methylation marks at the Pol-I-bound rDNA using sChIP approach in normal and KDM4A-depleted cells. Since histone H3 occupancy at the rDNA gene changed during activation (Supplementary Fig. 6b–d), signals obtained for methylated H3K9 marks were normalized to total H3 signal. No specific signal could be observed for H3K9me2 at rDNA loci (Supplementary Fig. 9b,e). However, we found that the level of H3K9me3 occupancy at the transcriptionally competent rDNA decreased upon serum refeeding whereas H3K9me1 occupancy increased (Fig. 6b,c), correlating spatially and temporally with the association of KDM4A with rDNA (Fig. 6a). Importantly, siRNA-mediated depletion of KDM4A prevented these changes (Fig. 6d,e; see also Supplementary Fig. 9 for non-normalized raw ChIP data). We were unable to detect any enrichment in H3K36me3 (another known substrate of KDM4A) at euchromatic rDNA (Supplementary Fig. 10).

Taken together, these results suggest that KDM4A specifically demethylates trimethylated H3K9 at the promoter and the transcribed regions of active rDNA repeats upon refeeding of starved cells and this demethylation plays an important role in the serum-mediated activation of rDNA transcription.

### PI3K pathway controls nucleolar recruitment of KDM4A

**Figure 7:**
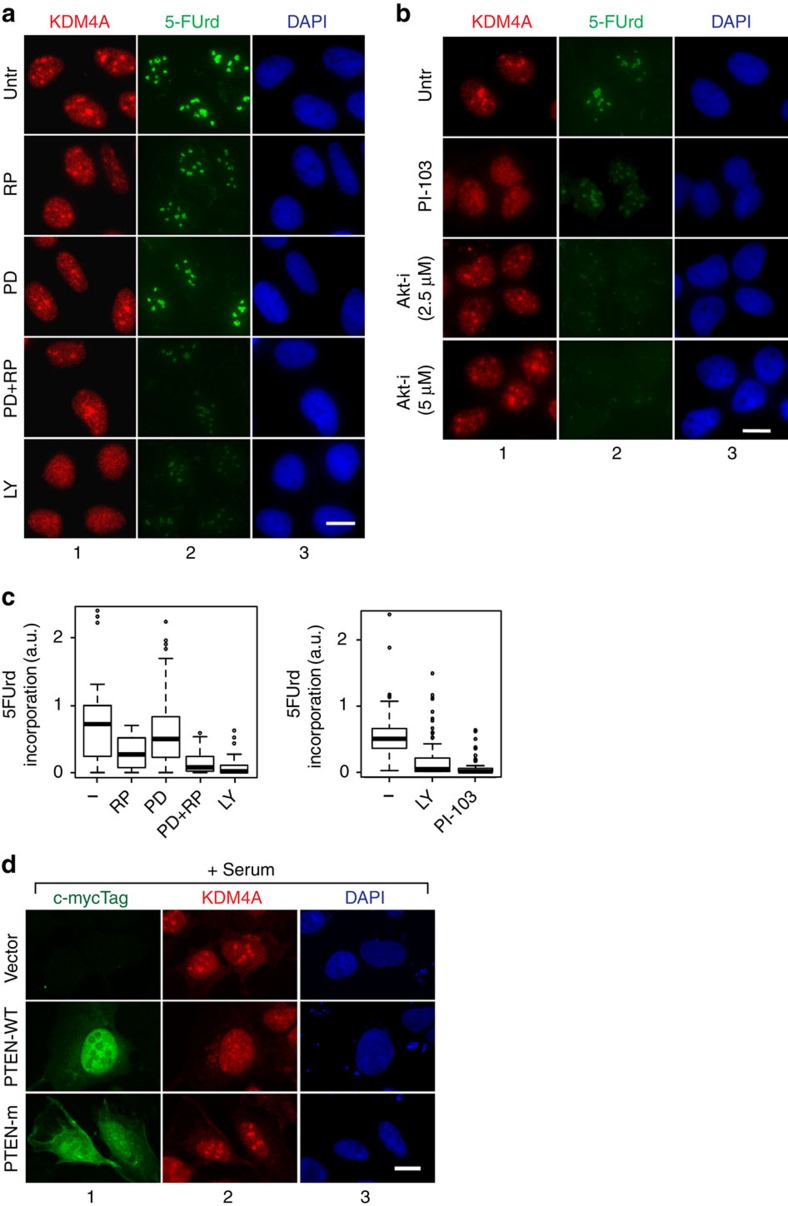
PI3K pathway controls nucleolar localization of KDM4A. (**a**) U2OS cells were serum starved for 24 h. Starved cells were incubated for 30 min with MAPK inhibitor PD98059 (PD), mTOR inhibitor rapamycin (Rapa), or PI3K inhibitor LY294002 (LY) or a mixture of mTOR and MAPK inhibitors (PD+Rapa) or DMSO as a control (Untr) and refed with serum in the presence of 5-FUrd and the respective inhibitors. Thirty minutes after serum addition cells were fixed and analysed by indirect immunofluorescence using antibodies specific to human KDM4A (Ab1, panels 1) and 5-FUrd (panels 2); nuclear DNA was stained by DAPI (panels 3). Scale bar, 5 μM. (**b**) U2OS cells were serum starved for 24 h. Starved cells were incubated for 30 min with PI3K inhibitor PI-103, AKT inhibitor AKTVIII (AKT-i), or DMSO (Untr) and refed with serum in the presence of 5-FUrd and the respective inhibitors. Thirty minutes after serum addition cells were fixed and analysed by indirect immunofluorescence using antibodies specific to human KDM4A (Ab1, panels 1) and 5-FUrd (panels 2); nuclear DNA was stained by DAPI (panels 3). (**c**) Quantification of 5-FUrd staining shown in **a** and **b** done as in Fig. 2e. The effect of inhibitors or combination of inhibitors are all significant (*P* value<10^4^, Wilcoxon test), except treatment with PD alone. Note that no incorporation could be measured upon AKT inhibition. The median values are shown as horizontal lines, outliers are shown as open circles; *n*=3. (**d**) U2OS cells were transfected with an expression vector encoding c-myc-tagged PTEN either wild-type (PTEN-WT) or mutated for its lipid phosphatase activity (PTEN-m). 24 h following transfection, cells were serum starved for 24 h and refed with serum containing 5-FUrd. Cells were fixed and analysed by indirect immunofluorescence using antibodies specific to c-myc Tag to detect overexpressed PTEN (panels 1); KDM4A (panels 2); nuclear DNA was stained by DAPI (panels 3).

PI3K, MAPK and mTOR pathways are three main pathways involved in the regulation of rDNA transcription in response to environmental cues^5^,^6^. We thus intended to characterize the role of these pathways in the control of KDM4A nucleolar localization. We treated cells with specific inhibitors of these kinases before serum stimulation and examined cellular localization of KDM4A and level of nascent rRNA synthesis (by 5-FUrd incorporation). The efficiency of inhibitors for their capacity to block phosphorylation of specific downstream targets was controlled by western blot (Supplementary Fig. 11). As expected we found that all inhibitors affected 5-FUrd incorporation and therefore rRNA synthesis at various levels (Fig. 7a,b, column 2, and c). However, only the PI3K inhibitors (LY29002 and PI-103), but not MEK/MAPKK (PD98059) or mTOR (Rapamycin) inhibitors, prevented the accumulation of KDM4A in the nucleolus upon serum addition (Fig. 7a,b, column 1; see Supplementary Fig. 11 for the effects of these inhibitors in serum deprived cells). Importantly, overexpression of wild-type PTEN, a PI3P phosphatase and as such a specific inhibitor of the PI3K pathway also blocked KDM4A relocalization to the nucleolus in response to serum (Fig. 7d), further demonstrating the involvement of the PI3K pathway in this subnuclear distribution of KDM4A.

The inhibition of MAPK or mTOR pathways affects 5-FUrd incorporation and therefore rDNA transcription to a lesser extent than the inhibition of PI3K pathway (Fig. 7a, column 2 and c). Thus, the loss of KDM4A localization followed by PI3K inhibition could be an indirect effect of severe repression of Pol-I transcription. To rule out this hypothesis, we combined the inhibition of the MAPK and mTOR pathways, which are known to cooperate in the serum-induced activation of rDNA transcription^23^. Such a treatment led to a blockage of rRNA production nearly as efficient as inhibition of PI3K pathway (Fig. 7a, column 2 and c). Nevertheless, it still had no effect on KDM4A localization (Fig. 7a, column 1). Thus, PI3K is the master regulator of KDM4A recruitment to the nucleoli.

Since we showed that KDM4A protein levels increase upon serum refeeding, we tested if PI3K, mTOR and MAPK pathway can regulate the stability of KDM4A. Our results, shown in Supplementary Fig. 5d,e, strongly suggest that it is not the case. Importantly, we found that PI3K inhibition does not affect serum-mediated induction of KDM4A expression levels (or its stability, see Supplementary Fig. 5d,e) while drastically inhibiting its nucleolar localization (Fig. 7), implying that nucleolar localization of KDM4A and its stabilization are independent serum-induced events.

Strikingly, control of KDM4A localization is independent on the classical PI3K–Akt pathway, since inhibition of Akt, a kinase activated by PI3K phospholipids products did not impede KDM4A relocalization to the nucleoli in response to serum (Fig. 7b, column 1), despite having a major inhibitory effect on rDNA transcription (Fig. 7b, column 2).

**Figure 8:**
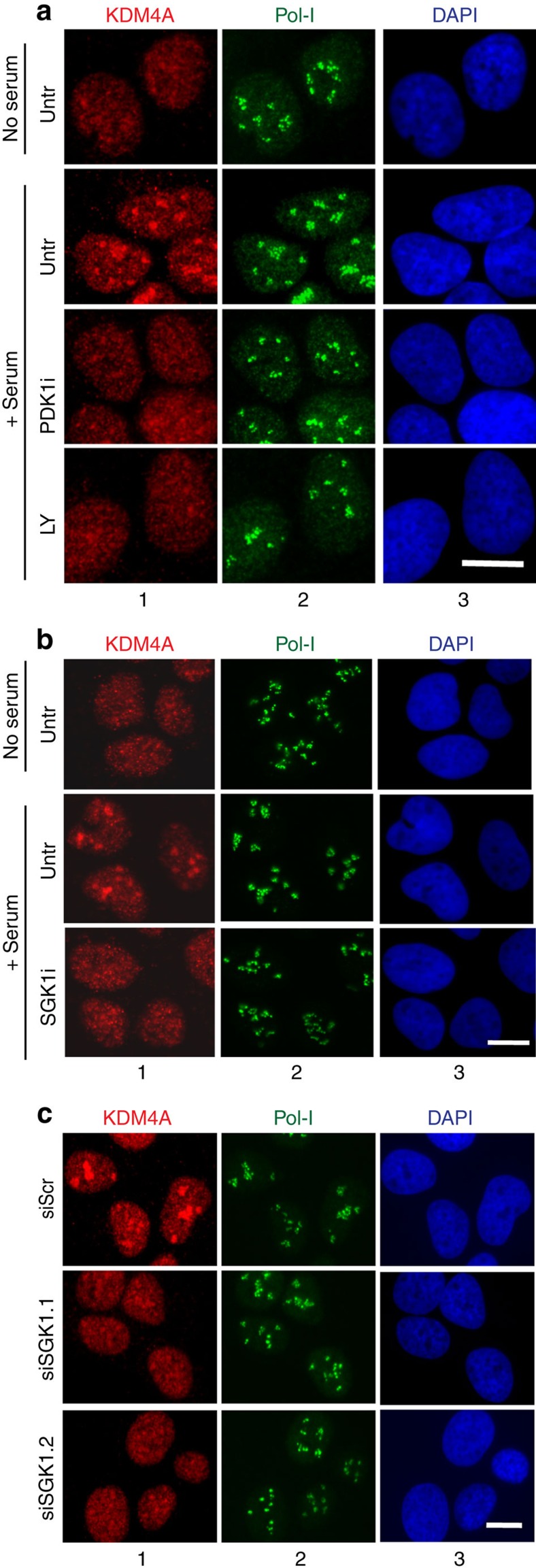
PI3K/SGK1 signalling cascade controls nucleolar localization of KDM4A. (**a**) U2OS cells were serum starved for 24 h. Starved cells were incubated for 30 min with PDK1 inhibitor GSK2334470 (PDK1i), or PI3K inhibitor LY294002 (LY) or DMSO as a control (Untr) and refed with serum in the presence of 5-FUrd and the respective inhibitors (+serum) or left starved (no serum). Thirty minutes after serum addition cells were fixed and analysed by indirect immunofluorescence using antibodies specific to human KDM4A (panels 1) and Pol-I (panels 2); nuclear DNA was stained by DAPI (panels 3). Scale bar, 5 μM. (**b**) U2OS cells were serum starved for 24 h. Starved cells were incubated for 30 min with SGK1 inhibitor GSK650394 (SGK1i), or DMSO as a control (Untr) and refed with serum in the presence of 5-FUrd and inhibitor (+serum) or left starved (no serum). Thirty minutes after serum addition cells were fixed and analysed by indirect immunofluorescence using antibodies specific to human KDM4A (panels 1) and Pol-I (panels 2); nuclear DNA was stained by DAPI (panels 3). Scale bar, 5 μM. (**c**) U2OS cells were electroporated with either a non-targeting siRNA (siScr) or two siRNAs directed against SGK1 (siSGK1-1 and siSGK1-2) and kept in serum-free medium for further 24 h before serum refeeding in the presence of 5-FUrd. Thirty minutes after serum addition, cells were fixed and analysed by indirect immunofluorescence using antibodies specific to human KDM4A (panels 1) and Pol-I (panels 2); nuclear DNA was stained by DAPI (panels 3). Scale bar, 5 μM.

We thus investigated kinases downstream of PI3K, which could be important for KDM4A relocalization. One attractive hypothesis was the involvement of the SGK1 kinase, known to regulate biological activities in a manner similar to Akt, and activated like Akt by PDK1, the major downstream effector kinase of PI3K^24^. Indeed, chemical inhibition of PDK1, as well as SGK1 (ref. 25) efficiently blocked KDM4A accumulation in the nucleolus co-stained for the presence of Pol-I (Fig. 8a,b, columns 1, see Supplementary Fig. 11a–d for western blots monitoring the efficiency of these inhibitors). Pol-I staining show that the integrity of nucleoli is preserved in inhibitor-treated cells. To confirm the involvement of SGK1 kinase, we inhibited its expression using two independent siRNAs (see Supplementary Fig. 11e for their validation), and we found that these two siRNAs efficiently blocked KDM4A nucleolar accumulation (Fig. 8c, column 1). Importantly, these inhibitors and siRNAs also blocked rDNA transcription as shown by 5-FUrd incorporation (Supplementary Fig. 7). We have summarized effect of inhibition/depletion of all investigated kinases on serum-driven activation of rRNA synthesis and relocalization of KDM4A in Table 1.

In summary, our results indicate that KDM4A accumulation in the nucleolus in response to serum stimulation is regulated by the PI3K–SGK1 signalling cascade, participating in the activation of rRNA synthesis.

## Discussion

Here, we present evidence that KDM4A is important for sustained activation of rDNA transcription in response to serum, since (i) depletion of KDM4A by specific siRNAs leads to the decrease of serum-stimulated rRNA synthesis observed both at the cell population levels and in individual cells (Fig. 3); (ii) this effect is complemented by the expression of siRNA-insensitive recombinant KDM4A (Fig. 5); and (iii) KDM4A is recruited to the rDNA during transcriptional activation (Figs 4 and 6). Notably, KDM4A is not essential for rDNA transcription: quantitative experiments relying on metabolic labelling indicate that KDM4A depletion resulted in a two- to threefold decrease in serum-induced rDNA transcription (Fig. 3). Moreover, serum-starved cells readily transcribe rDNA despite we did not find any evidence for KDM4A presence at the rDNA promoter (Fig. 4b). Furthermore, depletion of KDM4A from normally growing cells has no major effect of rDNA transcription level (Fig. 2). Thus, these data imply that KDM4A is required for efficient activation of rDNA transcription when cells have to recover a full level of rRNA production after experiencing unfavourable growth conditions (starvation in our case). However, this role is still very important, since even 50% reduction in rDNA transcription would have a significant impact on the capacity of cells to grow and divide (we have preliminary evidence from several approaches to suggest that ∼50% decrease in Pol-I transcription can slow cell proliferation up to six- to sevenfold). Furthermore, it has been demonstrated that maintenance of elevated levels of Pol-I activity in cancer cells appears critically important for the process of malignant transformation and cancer cell survival, as for example, a ∼35% reduction in Pol-I transcription in Eμ-Myc lymphoma cells resulted in induction of apoptosis as a consequence of immediate nucleolar stress, even before ribosome insufficiency takes effect on biosynthetic capacity^26^.

Our data also bring important insights into the precise step of rDNA transcription which requires KDM4A. Recent studies have revealed that in mammals, rDNA chromatin exists in three different epigenetic states^7^,^8^,^14^.

1. Firstly, silent, heterochromatic rDNA repeats which are characterized by high level of DNA methylation and repressive histone marks (40–60% of all repeats in cancer cells).
2. Secondly, poised, transcriptionally competent, euchromatic rDNA which is largely unmethylated and marked by hypoacetylated H3 and H4. The euchromatic poised rDNA genes harbour the bivalent marks H3K4me3 and H3K27me3, as well as trimethylated H3K9 (8,14 and this work). The so-called bivalent promoters are characteristic of genes that have to be quickly responsive to environmental cues, and have been first described for key development genes transcribed by Pol-II (ref. 27). Interestingly, Pol-I is present at poised rDNA repeats prior stimulation upon environmental changes (21 and our data).
3. Thirdly, active, transcribed euchromatic rDNA which is also unmethylated and characterized by higher levels of H3 and H4 acetylation combined with euchromatic histone marks.

The ratio between heterochromatic and euchromatic repeats does not change during the cell cycle or in response to environmental stimuli^10^,^28^, but can be altered during development or malignant transformation^19^. In contrast, the ratio between poised and transcribed rDNA repeats is regulated through epigenetic-based mechanisms allowing the adaptation of rDNA transcription during the cell cycle or in response to metabolic and environmental conditions (serum starvation/refeeding in this study). Therefore, unfavourable growth conditions lead to accumulation of poised rDNA repeats which are converted to their active form when conditions become more favourable. The molecular mechanisms that drive this conversion are not completely defined and this work identify KDM4A as an enzyme which is potentially involved in the initial stages of chromatin ‘activation'.

This transition indeed requires conversion of H3K9me3 to H3K9Ac. Here we propose that initial stage of this conversion is controlled by KDM4A which is recruited to rDNA shortly after refeeding (Figs. 4b and 6a). KDM4A targets H3K9me3 (a known substrate of this enzyme) at the rDNA promoter, allowing its demethylation and activation of rDNA transcription. The amount of H3K9me1 is inversely correlated to H3K9me3 upon KDM4A inhibition, suggesting that KDM4A specifically targets H3K9me3, converting it to H3K9me1.

However, further conversion to unmethylated H3K9, allowing its acetylation, must take place. Strikingly, it has been shown that the histone demethylase PHF8/JHDM1F associates with transcribed rDNA and positively regulates transcription by targeting H3K9me2/1 (refs 29, 30). We propose that KDM4A, at the initial stage of activation, removes H3K9me3 mark converting it to mono- or di-methylated K9, which can serve as substrates for PHF8, then allowing its acetylation and maximal activation of rDNA genes. In agreement with such a dynamic action of KDM4A and PHF8, both KDM4A and PHF8 bind to H3K4me3, the histone mark specific for active rDNA promoters^29^,^31^, which may explain stable association of both enzymes with the promoter and only transient association of KMD4A with transcribed region of rDNA upon refeeding (Fig. 4b).

Notably, our data suggest that KDM4A, although important, does not account for full transcriptional activation of rDNA in response to serum. Indeed, depletion of KDM4A seems to have more effect on H3K9me3 to H3K9me1 conversion (Fig. 6), than on rRNA transcription (between two- and threefold) (Fig. 3). One possible explanation is that only a subset of rDNA repeats is regulated by H3K9 methylation and KDM4A, whereas others escape such regulation. The presence of significant level of basal rRNA transcription in starved cells and our data on steady-state level of transcription (Fig. 2) support this explanation. An alternative, but not mutually exclusive hypothesis could be that compensatory mechanisms are capable to induce a hyper-activation of the small fraction of rDNA repeats that have lost H3K9me3 in KDM4A-depleted cells.

Finally, we can conclude that at euchromatic rDNA, we observe a dynamic picture of ever-changing histone modifications which play an essential role in the flexibility of transcriptional regulation but the full complexity of which is still not entirely understood.

Another exiting finding of this study is the discovery of a link between the control of the subcellular localization of an enzyme involved in epigenetic control of transcription (KDM4A) and the activity of a signalling cascade regulated by the availability of nutrients and growth factors (PI3K).

Three major pathways (PI3K, mTOR and MAPK) are activated in response to nutrients/growth factors and known to regulate rDNA transcription^6^,^32^,^33^. PI3K is a prominent signalling cascade involved in control of cell growth, proliferation and survival and that is often upregulated in various cancers^34^. PI3K is known to regulate Pol-I transcription alone or in conjunction with mTOR and MAPK pathways^6^,^23^. Many signalling cascades including PI3K pathway, have been shown to target chromatin-modifying enzymes including histone demethylases, affecting either their stability, localization or activity (for reviews see refs 35, 36, 37). The question of how KMTs/KDMs are linked to signalling pathways is challenging and only few examples have been documented. KMTs/KDMs are targeted by signalling factors inside the nucleus leading in changes in partner association, targeting to chromatin and/or substrate specificity^38^,^39^. Here, we found that the PI3K pathway (but not the mTOR or MAPK pathways) controls KDM4A cellular localization and therefore its recruitment to rDNA through its downstream kinase SGK1. Indeed, inhibition of PI3K or SGK1 suppress both KDM4A relocalization to nucleoli of refed cells and activation of Pol-I transcription (Figs 7 and 8). Our data also suggest the involvement of the PDK1 kinase, which links PI3K and SGK1, accordingly to its described role in SGK1 activation, downstream of PI3K^24^,^40^.

**Figure 9:**
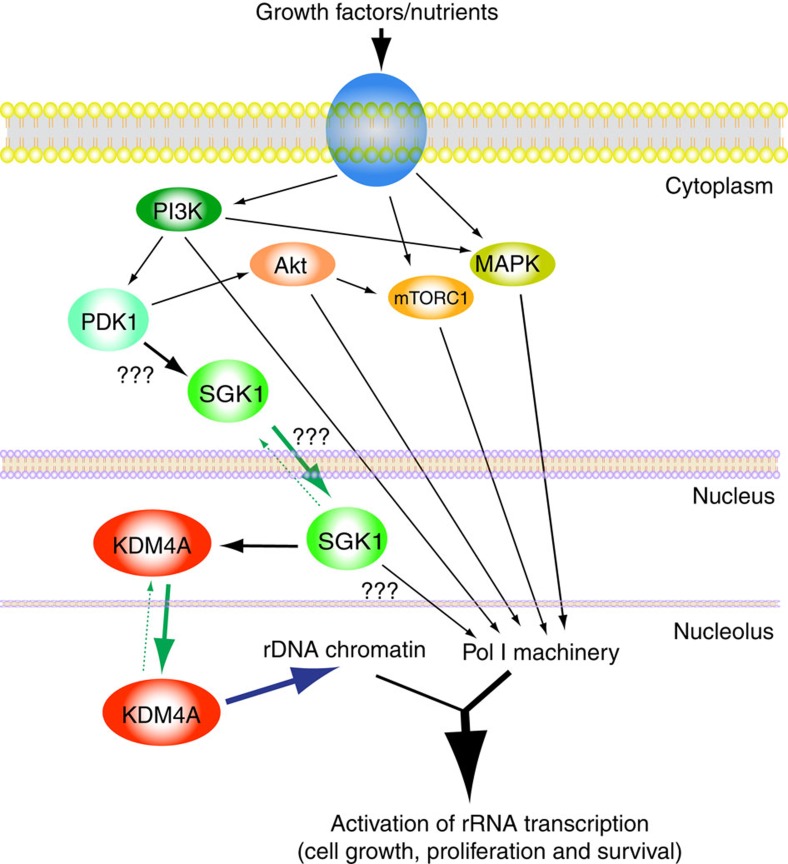
Schematic outline of serum-induced pathways controlling rDNA transcription. PI3K–Akt, mTOR and MAPK pathways regulate rRNA gene transcription by targeting RNA Polymerase I machinery (as previously published, see Discussion). PI3K–PDK1–SGK1 signalling cascade regulates activation of rRNA gene transcription by controlling histone modifications at rDNA *loci* through KDM4A localization. SGK1 may in addition target Pol-I transcription machinery independently of KDM4A.

Our finding represents to our knowledge the first example of the regulation of the intranuclear partition of a KDM between the nucleoplasm and the nucleolus, which is required to adapt rDNA transcription, and consequently ribosome biogenesis, to the availability of growth factors/nutrients. The PI3K signalling pathway is known to regulate rDNA transcription by targeting various components of Pol-I transcription machinery. In response to serum/nutrients stimulation it regulates both TIF-1A/RRN3 stability and nucleolar localization through Akt-mediated modulation of CK2 activity^41^. PI3K was also described to directly phosphorylate UBF^42^ and regulate SL1 occupancy at the promoter^23^. PTEN, a negative regulator of the PI3K pathway was described to inhibit Pol-I transcription by disturbing the SL1 complex on rDNA, although the mechanism remains unclear^43^. Our data add another mechanism for the regulation of rDNA transcription by PI3K that takes place at the chromatin level through the regulation of KDM4A by PI3K–SGK1 signalling cascade. Hence, the distinct routes converge controlling the activation of rDNA transcription (see model of the regulation of rDNA by PI3K in Fig. 9).

What is the mechanism by which the PI3K–SGK1 cascade favours nucleolar accumulation of KDM4A? Interestingly, we found that serum treatment leads to increased stability of KDM4A and consequently its accumulation (Supplementary Fig. 5). However, this is unlikely to be linked to KDM4A relocalization, since PI3K inhibition does not prevent the serum-induced increase in KDM4A level (Supplementary Fig. 5) but blocks its nucleolar shuttling (Fig. 7). Thus, the PI3K–SGK1 cascade regulates the affinity of KDM4A for the nucleolus, most probably by phosphorylating KDM4A (or its associated partner), thus facilitating its nucleolar translocation (see our model in Fig. 9). Interestingly, SGK1 inhibition/depletion has a very strong impact on rRNA synthesis, which is comparable to the effect of PI3K inhibition (Supplementary Fig. 7). This suggests that like PI3K, SGK1 may potentially regulate Pol-I transcription by targeting various components of Pol-I transcription apparatus. Therefore, SGK1 may have a dual role in the regulation of Pol-I transcription: (i) as shown here, it regulates KDM4A cellular localization on serum-dependent manner affecting as consequence rDNA chromatin structure and (ii) in addition, it could target components of Pol-I transcription machinery either directly or indirectly, thus further enhancing the rDNA transcriptional activation. Clearly, further studies are required to determine how exactly the PI3K/SGK1 axis regulates KDM4A localization and Pol-I transcription.

In conclusion, here we propose a novel role of PI3K signalling in the regulation of epigenetic changes at specific loci. PI3K acts through SGK1 by regulating the subcellular localization of histone demethylase KDM4A in response to serum. KDM4A moves inside the nucleolus to target trimethylated H3K9, initiating events leading to the activation of rDNA transcription. Interestingly, strong links have been uncovered between upregulation of Pol-I transcription or PI3K activity and cancer^34^ and now we have established that KDM4A is targeted by PI3K pathway and controls activation of Pol-I transcription. Our results thus extend a general role of KDM4A in the regulation of protein synthesis and imply that KDM4A may be a very promising target in anticancer therapy.

## Methods

### Cell culture conditions

U2OS cells were obtained from ATCC and maintained according to the supplier's instructions in McCoy's 5A medium plus 10% FBS (PAA). For serum starvation–refeeding experiments cell were grown to ∼60–70% confluency, washed twice with Dulbecco's PBS and then serum-starved for 20–24 h in DMEM low glucose (1 g l^−1^). For activation of Pol-I transcription, serum-starved cells were incubated in DMEM low glucose (1 g l^−1^) containing 20% FBS (Lonza). All media were supplemented with 100 U/ml penicillin and 100 μg ml^−1^ streptomycin (Gibco) and cells were maintained in a 37 °C incubator with 5% CO_2_. Hela cells were obtained from ATCC and grown in DMEM containing 10% FBS. GC32 cells were a gift from Bernard Lopez (Toulouse, France) and WI38 cells were given by Estelle Nicolas (Toulouse, France).

The final concentration of kinase inhibitors dissolved in DMSO was as described elsewhere: PD98059 (25 μM); Rapamycin (50 nM); LY29002 (20 μM); PI-103 (5 μM); AktVIII (2.5 or 5 μM); GSK2334470 (1 μM); GSK650394 (10 μM). They were added to cells 1 h before refeeding and kept afterwards. Cycloheximide (Sigma) was used at 100 μM as previously described for the study of KDM4A stability in U2OS cells.

### Transfection

Wild-type human HA-tagged KDM4A CMV-driven expression plasmid was a gift from J. Christensen. The demethylase-dead KDM4A mutant was constructed by mutating H188 to A, directly in the WT plasmid using the quick change mutagenesis kit following the manufacturer's instructions (Agilent Technologies). siRNA were purchased from Eurogentec and are described in Supplementary Table 3. For transfection, 10^6^ U2OS cells were electroporated with double-stranded siRNA to a final concentration of 1 μM, separately or together with 2 μg of plasmids by using an eletroporation device (Amaxa AG, Cologne, Germany) according to the manufacturer's specifications (Buffer V, X001 program). Expression plasmid encoding myc-tagged wild-type PTEN (pcDNA3-PTENWT) was a gift from Evelyne Goillot (ENS Lyon, France). Cells were seeded on glass coverslips and transfected with either empty pCDNA3, pcDNA3-PTENwt or pcDNA3-PTENmut using jetPEI with a ratio of 1 μg DNA for 2 μl of jetPEI following the manufacturer's recommendation (Ozyme).

### RNA labelling in cells and rRNA analysis

Labelling of RNA in cells (∼70% confluent or ∼50% for starved/refed) and RNA analysis was performed essentially as described^44^ using 10 mCi ^3^H-uridine for ∼0.2–0.4 × 10^5^ cells per well of a six-well plate. RNA was extracted (Pure Link RNA Mini kit, Life Technologies). 2 μg of ^3^H-labelled total RNA was run on a 1% formaldehyde agarose gel at 120 V for 95 min in 1 × MOPS, blotted onto Hybond-N membrane (Amersham), crosslinked (ultraviolet cross-linker; UVP), analysed by tritium imaging using Fuji Tritium image plate (or following PerkinElmer En^3^Hance spray, exposed to Kodak Biomax XAR film at −80°C) and then quantified using Aida software. The s.d. was calculated from three independent experiments.

*In situ* RNA labelling was performed using 5-Fluorouridine (5-FUrd, Sigma). Cells were incubated with 1 mM 5F-Urd for 30 min before fixation with 4% paraformaldehyde in PBS, for 15 min at 4 °C. 5-FUrd incorporation was analysed by immunofluorescence using anti-BrdU antibodies (BU-33, Sigma). All the staining procedure was performed at 4 °C, in the presence of 0.4 U ml^−1^ RiboGuard RNAse inhibitor (Epicentre). Quantification of fluorescence levels was done on ∼100–200 cells per slide using home-developed macros in Image J software (National Institutes of Health) and analysed using the R open source software R Core Team (2014), (http://www.R-project.org/).

### ChIP and sChIP assays

Cells were grown until 70% confluent, crosslinked with formaldehyde (final concentration 1%) for 10 min and the cross-linking was stopped by addition of glycine (final concentration 0.125 M) for 5 min. Crosslinked chromatin was shared using Biorupter (Diagenode) to 300-base-pair average size. For single round ChIP, immunoprecipitation was carried out using chromatin isolated from 1 × 10^6^ cells, antibodies listed in Supplementary Table 1 or appropriate control IgGs and 30 μl of Protein A/Protein G magnetic beads (Life Technologies). For sChIP, first round of immunoprecipitation was carried out using chromatin isolated from ∼7 × 10^6^ cells, 200 μg RPA135 antibody and 200 μl of Protein A/Protein G magnetic beads (Life Technologies). All antibodies used are listed in Supplementary Table 1. Chromatin was eluted from beads using two steps elution. Initially beads were incubated overnight at 4 °C in 400 μl of re-ChIP elution buffer (1% Triton X-100, 2 mM EDTA, 150 mM NaCl, 20 mM Tris-HCl, pH 8.1) containing 80 μg of RPA135 (N-17) P peptide (Santa Cruz). Next, beads were resuspended in 100 μl of 10 mM DTT in re-ChIP elution buffer and incubated 30 min at 37 °C. Eluates from both steps were pooled, and volume was adjusted to 5 ml by ChIP IP buffer (1% Triton X-100, 0.1% Sodium Deoxycholate, 0.1% SDS, 1 mM EDTA, 125 mM NaCl, 20 mM Tris-HCl, pH 7.5). Second round of immunoprecipitation was carried out using 1 ml of chromatin from the first round (equivalent of∼1.4 × 10^6^ cells), antibodies listed in Supplementary Table 1 or appropriate control IgGs and 30 μl of Protein A/Protein G magnetic beads (Life Technologies). Purified immunoprecipitated DNA was analysed by two tetraplex qPCR panels designed for 8 regions of the rDNA repeat; Promoter, IGS4, 18S, 5.8S and 28S, Terminator, IGS1 and 5′ETS (see Supplementary Table 2 for primers and probes sequences). Reactions were carried out on a LightCycler 480 with a reaction volume of 10 μl per well in triplicates. The specific signals were calculated as the difference between the signals from the specific antibody (that is, H3) and from the negative control (an appropriate IgG) and were expressed as percentage of input chromatin. When mentioned the specific signals of modified histone H3 were normalized to the specific H3 signals with the standard deviation calculated from three independent ChIP experiments. Signal representing the TrR is the average of the combined signal from 5′ETS, 18S, 5.8S and 28S rRNA. Signal representing the nTrR is the average of the combined signal from IGS1 and IGS2.

### Immunofluorescence

Cells were fixed with 4% paraformaldehyde and permeabilized with 1% Triton X-100 in PBS or permeabilized 5 min at 4° with 1% Triton X-100 before fixation with 4% paraformaldehyde as indicated in figure legends. Cells were then incubated at 4 °C overnight with the indicated primary antibodies: Rabbit polyclonal anti-JMJD2A (Bethyl A-300-861-A; 0.5 μg ml^−1^), Rabbit polyclonal anti-KDM4A antibodies (Sigma, HPA007610, 0.5 μg ml^−1^), Rabbit polyclonal anti-HA (Cell Signaling C29F4, 1 μg ml^−1^), RPA 194 Santa Cruz SC-48385 1 μg ml^−1^, mouse monoclonal anti-UBF (SC-13125 1 μg ml^−1^), mouse monoclonal anti-BrdU 2 μg ml^−1^ (BU-33, Sigma), mouse monoclonal anti-myc antibodies (9E10, Santa Cruz). AlexaFluor-conjugated secondary antibodies were purchased from Fisher Scientific. Observations were carried out with a fluorescence microscope (DM5000; Leica, Wetlar, Germany) equipped with a cooled charge-coupled device camera, and images were acquired using the MetaMorph imaging system (Universal Imaging Corp., West Chester, PA). For Z-stacks acquisition (Fig. 1b), images were collected with a DM6000 Leica microscope equipped with semrock filters, a PiFoc piezofocus device (Physic Instrumente), and a CollSnapHQ2 camera (Roper Scientific), controlled by MetaMorph software (Universal imaging Corp.). Five Z-sections at 0.2 μm spacing were projected onto a single plan.

Box-and-whisker plots of quantification of 5-FUrd staining were obtained with the R open source software R Core Team (2014), (http://www.R-project.org/). They show the median, the 25 and 75% quantiles, as well as outliers (outliers are identified by not being in the range [25%Quantile−1.5 × InterQuantiles; 75%Quantile+1.5 × InterQuantiles] where interQuantiles=75%Quantile−25%Quantile). These representations have to be accompanied by statistical analysis of the comparison between the two populations. Statistical hypothesis tests were applied to confirm whether the hypothesis (that can be seen on the boxplot) that there is a differences between indicators of the two populations (such as mean, median, distribution) can be considered as true with a great confidence or can be due to random effect. Because data distribution was not normal (due to a threshold applied during quantification to remove background staining), we used a Wilcoxon test to reject the hypothesis that the two populations means are the same and thus conclude that there is a significant difference between the two means if the *P* value is<0.05, meaning a confidence of 95%.

### Immunoblotting and immunoprecipitation

For western blotting, cells were lysed in NuPage Sample lysis Buffer supplemented with reducing agent (Invitrogen) and heated for 5 min at 85 °C to denature proteins. Western blots were performed using standard procedures and antibodies were used at the following concentrations: rabbit anti-KDM4A 0.4 μg ml^−1^ (Bethyl A-300-861-A), rabbit anti-RPA194 0.2 μg ml^−1^ (Santa Cruz SC-48385), mouse anti-PAF53 0.25 μg ml^−1^ (BD Transduction Labs, # P95220), mouse anti-UBF 0.2 μg ml^−1^ (Santa Cruz SC-13125), mouse anti-α-tubulin 0.1 μg ml^−1^ (T6199, Sigma), rabbit anti p70S6K (Cell Signaling, #9202), rabbit anti-PhosphoS6K (Cell Signaling, #9205), anti-Akt (Cell Signaling, #9272), anti-Phospho-Akt (Ser473) (Cell Signaling, #9271), anti-Phospho-Erk1/2 (Cell Signaling, #9101), anti-NDRG1 (Cell Signaling, #9485); anti-Phospho-NDRG1 (Cell Signaling, #5482), anti-SGK1 (Cell Signaling, #12103). All Cell signaling Ab were diluted 1/1,000. Peroxidase-conjugated secondary antibodies were purchased from Amersham.

For immunoprecipitation, 10^7^ cells were resuspended in hypotonic buffer (10 mM Tris PH 7.4, 1 mM MgCl_2_, 10 mM KCl and 1 mM DTT). After breaking the cells with a Dounce B pestle, nuclei were spin down at 500 g for 10 min and lysed in IP buffer (10 mM Tris pH 8, 0.4% NP40, 300 mM NaCl, 10% glycerol, 1 mM DTT, anti-protease (Roche), anti-phosphatase (Sigma) inhibitors). Lysates were diluted using one volume of dilution buffer (10 mM Tris pH 8, 5 mM CaCl_2_, 2 U/ml RQ1 DNAse (Promega) and then incubated with rabbit anti-KDM4A antibodies 1 μg ml^−1^ (A-300-861-A, Bethyl) or rabbit anti-HA antibodies 1 μg ml^−1^ (C29F4 Cell signaling) as a control, overnight at 4 °C. Protein complexes were pull-down using a mix 1:1 of Protein A coupled to agarose (Sigma):Protein G coupled to sepharose (Sigma). Following several washes with IP buffer, beads were eluted with NuPAGE sample lysis buffer (Invitrogen) and analysed by western blot. All steps were performed at 4 °C.

### mRNA level analysis

Total RNA was extracted from cells by using the RNeasy mini kit (Qiagen). Five hundred nanograms of RNA were reverse transcribed for 50 min at 42 °C in a 20 μl reaction volume containing 0.5 μM dNTPs, 0.5 μg of random primers, 10 mM DTT, 1 × AMV RT buffer, 40 U of RNasin and 10 U of AMV Reverse Transcriptase (Promega). Samples were incubated for 15 min at 70 °C to stop the reaction. Samples were analysed by qPCR by using the primers described in Supplementary Table 4 on a CFX96 real-time system device (Biorad) using the Platinum SYBR Green qPCR SuperMix (Invitrogen).

## Additional information

**How to cite this article:** Salifou, K. *et al.* The histone demethylase JMJD2A/KDM4A links ribosomal RNA transcription to nutrients and growth factors availability. *Nat. Commun.* 7:10174 doi: 10.1038/ncomms10174 (2016).

## Supplementary Material

Supplementary Information Supplementary Figures 1-11 and Supplementary Tables 1-4

## Figures and Tables

**Table 1:** Effect of the inhibition of various serum-activated kinases on the activation of rDNA transcription and on the nucleolar localization of KDM4A.

**Inhibited kinase**	**How inhibited**	**rDNA transcription level**	**Nucleolar accumulation of KDM4A**
None	None	+	+
mTOR	Rapamycin	−	+
MEK/MAPK	PD98059	−/+	+
mTOR+MEK	Rapamycin+ PD98059	−	+
PI3K	LY29002 PI-103 overexpression of wt PTEN	−	−
PDK1	GSK2334470	−	−
AKT	AktVIII	−	+
SGK1	GSK650394 siRNA	−	−
