# Supplementary material for: The histone demethylase JMJD2A/KDM4A links ribosomal RNA transcription to nutrients and growth factors availability
Source: Nat Commun. 2016 Jan 5;7:10174. doi: 10.1038/ncomms10174 (PMC5157185; doi:10.1038/ncomms10174)
Supplement: Supplementary Information — Supplementary Figures 1-11 and Supplementary Tables 1-4 [file ncomms10174-s1.pdf]

Supplementary Figures

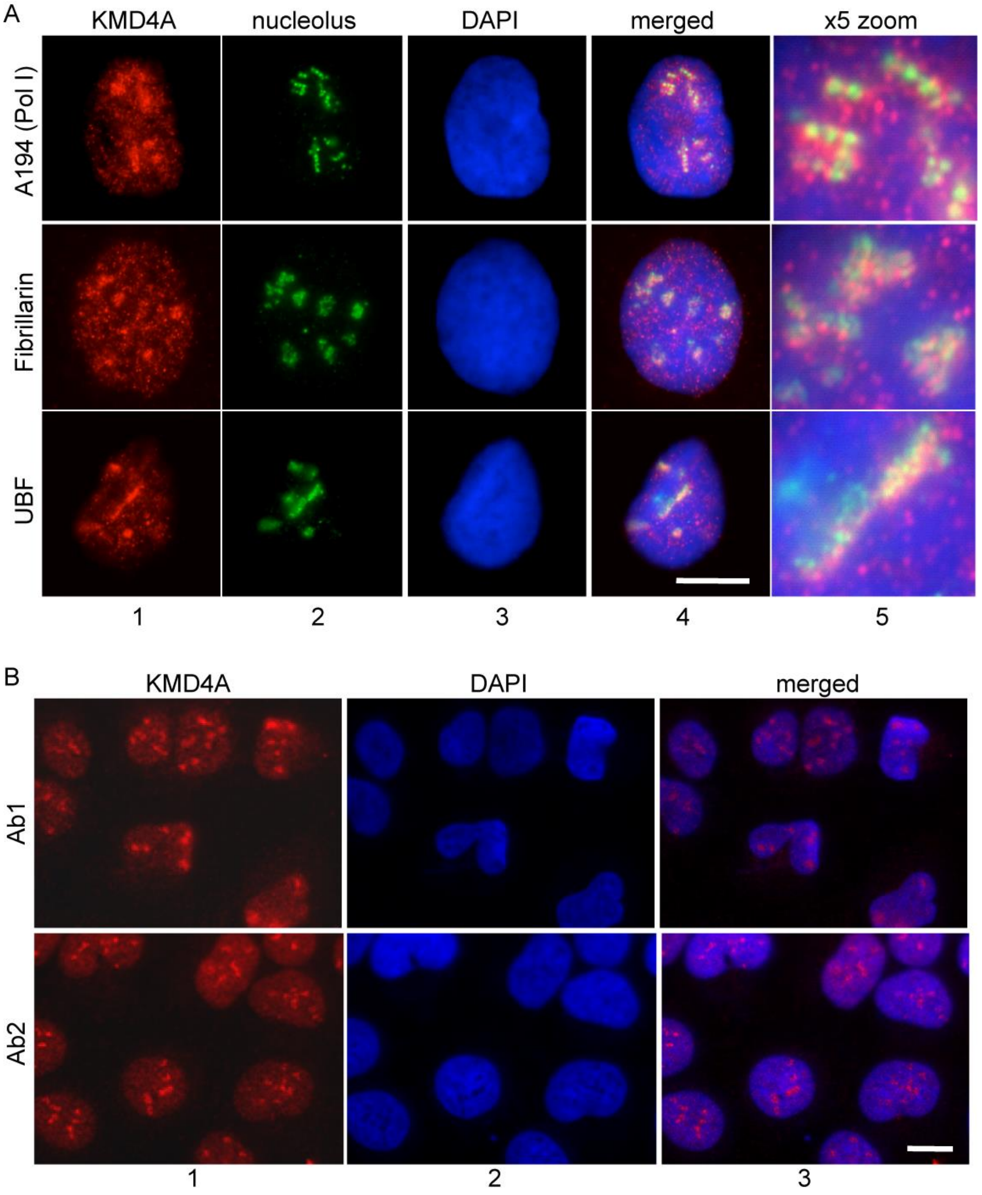

Supplementary Figure 1. **Co-localisation between KDM4A and nucleolar proteins.**
A. Actively growing U2OS cells (60-70% confluent) were fixed following extraction with 1% Triton X100 and analysed by indirect immunofluorescence using antibodies specific to human KDM4A (Ab1 Bethyl Lab, panels 1), and nucleolar markers (panels 2 (nucleolus), Fibrillarin, Pol-I largest subunit A194 and UBF from top to bottom). Nuclear DNA was stained by DAPI (panels 3). Images were acquired with a Leica microscope (DM6000, 100X Objective). 5 Z-sections at 0.2 μm spacing were projected onto a single plan. One representative nucleus with associated merged images (panel 4) and a 5 X zoom on nucleoli containing area (panel 5) are shown. Bar = 5 μM.
B. Actively growing U2OS cells (60-70% confluent) were fixed and analysed by indirect immunofluorescence using two distinct antibodies specific to human KDM4A (panel 1): Ab1

(Bethyl Lab) and Ab2 (Sigma). Nuclear DNA was stained by DAPI (panel 2). Merged images are shown (panel 3). Bar = 5 μM.

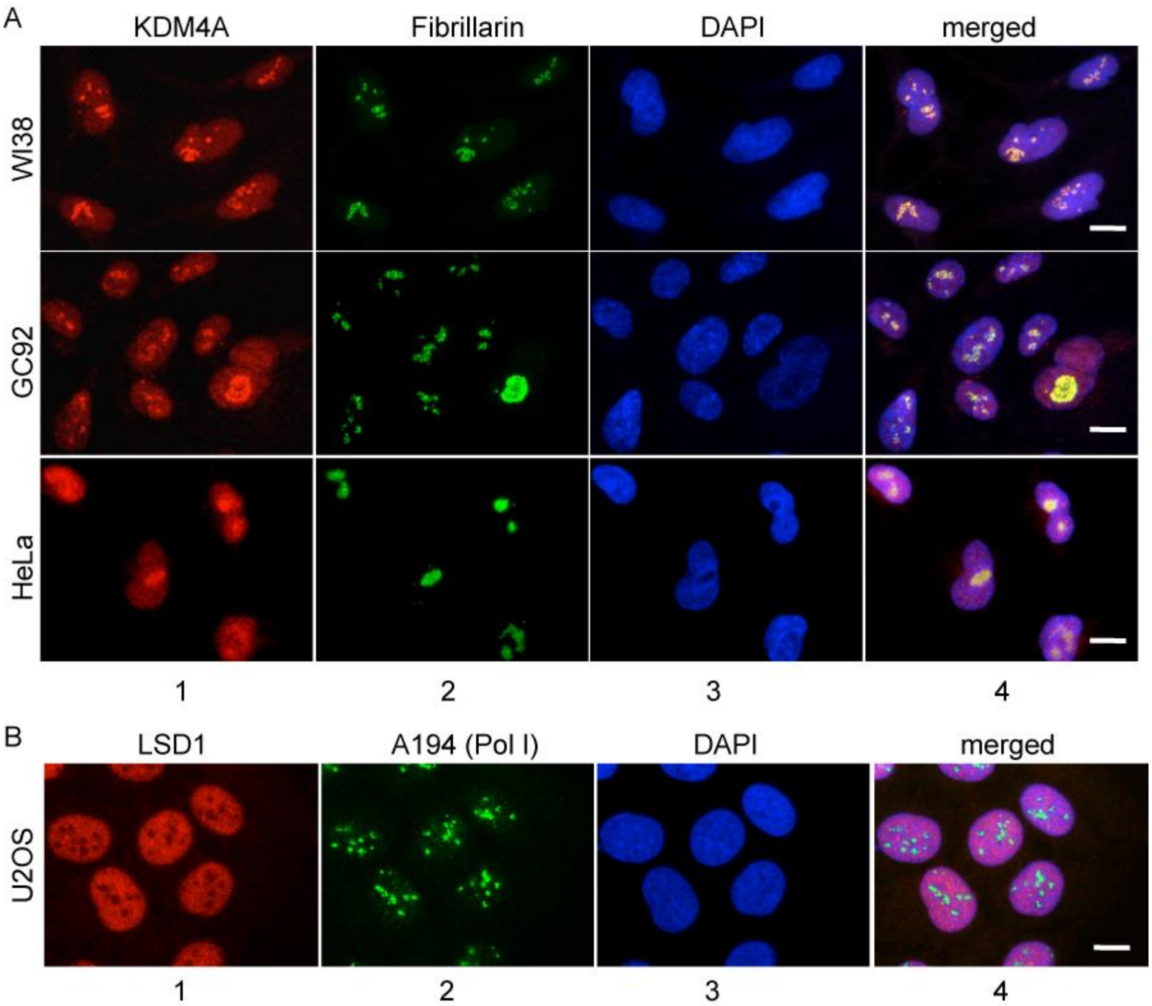

Supplementary Figure 2. KDM4A colocalisation with nucleolar protein in various cell lines.

A. WI38, GC92 and HeLa human cells were fixed and analysed by indirect immunofluorescence using antibodies specific to human KDM4A (Ab1 Bethyl Lab, panel 1) and Fibrillarin (panel 2); nuclear DNA was stained by DAPI (panel 3). Merged images are shown (panel 4). Bar = 5 μM.

B. U2OS cells were fixed and analysed by indirect immunofluorescence using antibodies specific to human LSD1 (panel 1), and human Pol-I largest subunit A194 (panel 2); nuclear DNA was stained by DAPI (panel 3). Merged images are shown (panel 4). Bar = 5 μM.

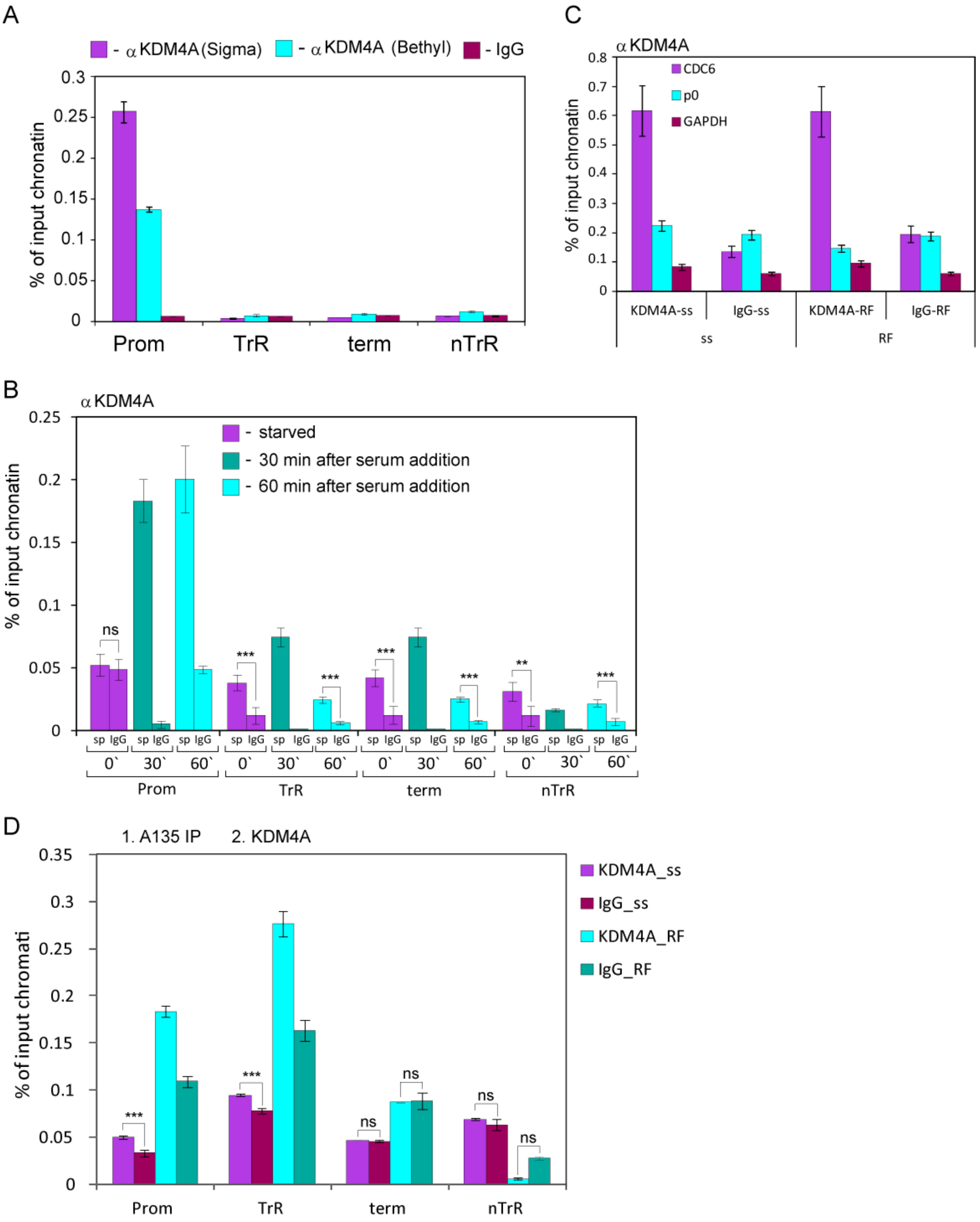

Supplementary Figure 3. ChIP raw data.

A. Raw data of ChIP assays performed in Fig. 1c. The value of each bar represents the signal either from the specific antibody or from the negative control (an appropriate IgG) expressed as

% from total chromatin input. Signal representing the transcribed region (TrR) is the average of the combined signal from 5'ETS, 18S, 5.8S and 28S rRNA. Signal representing the non-transcribed region (nTrR) is the average of the combined signal from IGS1 and IGS2. Standard deviations from three independent experiments are shown; n=3.

B. Raw data of ChIP assays performed in Fig. 4b. The value of each bar represents the signal either from the specific antibody or from the negative control (an appropriate IgG) expressed as % from total chromatin input. Signal representing the transcribed region (TrR) is the average of the combined signal from 5'ETS, 18S, 5.8S and 28S rRNA. Signal representing the non-transcribed region (nTrR) is the average of the combined signal from IGS1 and IGS2; standard deviations and statistical significance are shown; *** $p<0.001$; ** $p<0.01$; ns $p\geq0.05$. P-values have been calculated using one and two-way ANOVA on R software; $n = 3$.

C. Raw data of ChIP assays performed in Fig. 4c. The value of each bar represents the signal either from the specific antibody or from the negative control (an appropriate IgG) expressed as % from total chromatin input. Standard deviations from three independent experiments are shown; n=3.

D. Raw data of ChIP assays performed in Fig. 6a. The value of each bar represents the signal either from the specific antibody or from the negative control (an appropriate IgG) expressed as % from total chromatin input. Signal representing the transcribed region (TrR) is the average of the combined signal from 5'ETS, 18S, 5.8S and 28S rRNA. Signal representing the non-transcribed region (nTrR) is the average of the combined signal from IGS1 and IGS2; standard deviations and statistical significance are shown; *** $p<0.001$; ns $p\geq0.05$. P-values have been calculated using one and two-way ANOVA on R software. Note that we observe a considerable (and unexplained) increase in the level of IgG signal in refed cells; $n = 3$.

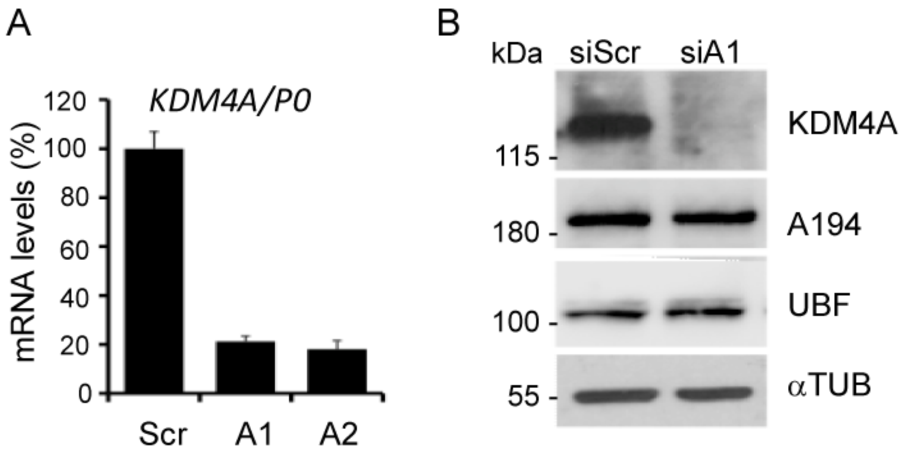

Supplementary Figure 4. Efficiency of siRNA mediated KDM4 depletion.
A. The levels of the mRNAs KDM4A were analysed in cells electroporated with either a non-targeting siRNA (Scr) or two siRNAs directed against KDM4A (A1 and A2). Signals were normalized to the mRNA levels of the housekeeping gene encoding the ribosomal phosphoprotein P0.
B. U2OS cells were electroporated with either a non-targeting siRNA (siScr) or a siRNA directed against KDM4A (siA1) and the levels of KDM4A, A194 and UBF were determined 24 hours post-transfection by Western-blotting; TUBULIN (α-TUB) was used as a loading control. Positions of molecular weight markers (PageRuller, Thermo Scientific) are indicated.

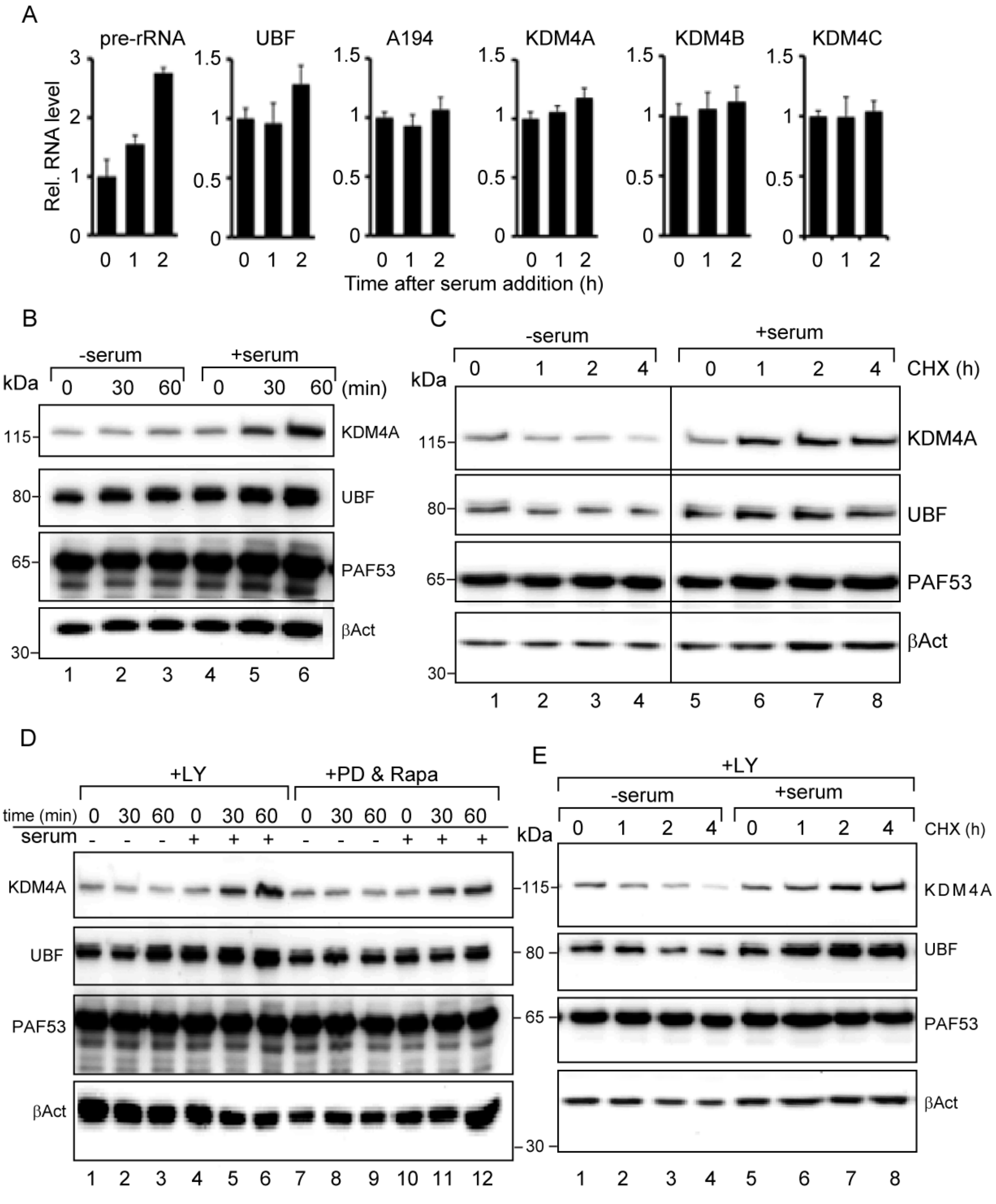

Supplementary Figure 5. KDM4A stability is regulated by serum independently from PI3K, mTOR and MAPK pathways.

A. U2OS cells were serum starved for 24 hours (t=0) and refed with serum. Cells were collected at 1 hour (t=1) and 2 hours (t=2) following serum addition. The levels of the 45S pre-rRNA, and

the mRNA encoding for UBF, A194, and KDM4A-C were analysed at each time point. Signals were normalized to the mRNA levels of the housekeeping gene P0.
B. U2OS cells were serum starved for 24 hours (t=0) and refed with serum or left starved, as indicated. Levels of KDM4A, Pol-I subunit PAF53 and UBF were determined in total cell extracts at different time points after re-feeding by Western-blotting; β-Actin (β-Act) was used as a loading control.
C. U2OS cells were serum starved for 24 hours and protein synthesis was inhibited by cycloheximide (CHX). Cells were either left starved (lanes 1-4) or treated with serum (lanes 5-8). Levels of KDM4A, Pol-I subunit PAF53 and UBF were determined in total cell extracts at different time points after cycloheximide treatment by Western blotting. β-Actin (β-Act) was used as a loading control.
D. U2OS cells were serum starved for 24 hours. Starved cells were incubated for 30 min with PI3K inhibitor LY294002 (LY) or a mixture of mTOR (rapamycin) and MAPK (PD98059) inhibitors (PD+Rapa) and refed with serum or left starved, as indicated (t=0). Levels of KDM4A, Pol-I subunit PAF53 and UBF were determined in total cell extracts at different time points after re-feeding by Western-blotting; β-Actin (β-Act) was used as a loading control.
E. U2OS cells were serum starved for 24 hours. Starved cells were incubated for 30 min with PI3K inhibitor LY294002 (LY). Protein synthesis was inhibited by cycloheximide (CHX). Cells were either left starved (lanes 1-4) or treated with serum (lanes 5-8). Levels of KDM4A, Pol-I subunit PAF53 and UBF were determined in total cell extracts at different time points after cycloheximide treatment by Western blotting. β-Actin (β-Act) was used as a loading control.

Positions of molecular weight markers (PageRuller Plus, Fermentas) are indicated.

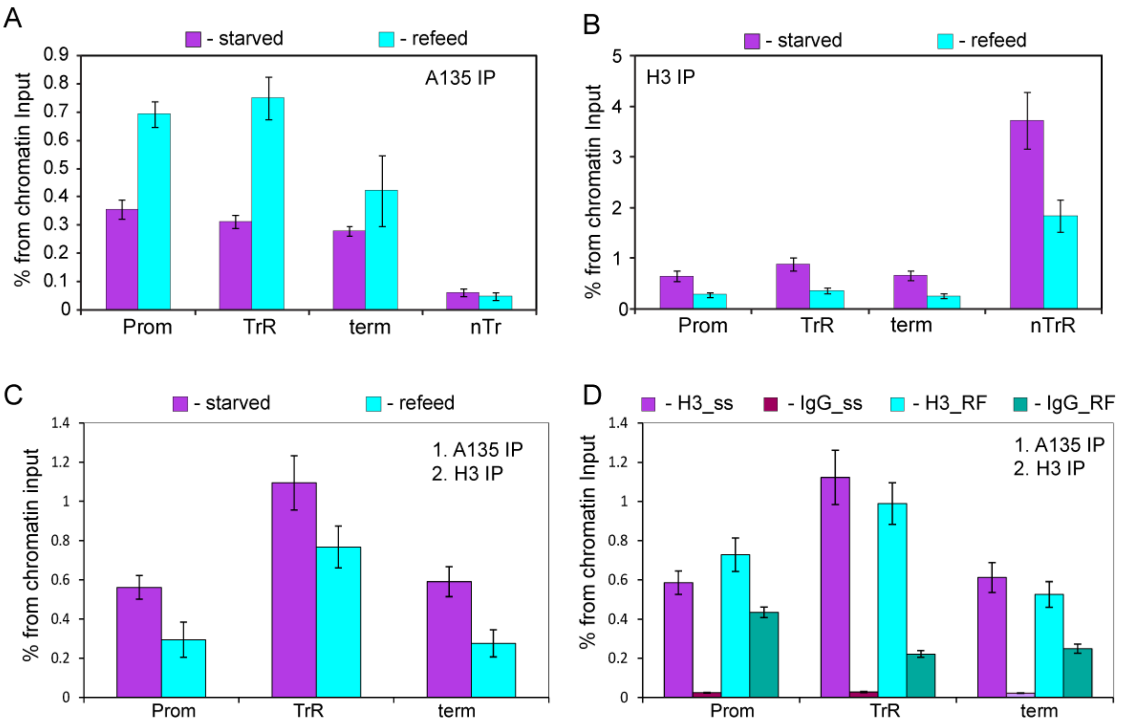

Supplementary Figure 6. Histone H3 occupancy is affected during activation of Pol-I transcription.

A. ChIP assays were performed using antibodies specific to human A135 (subunit of Pol-I) and chromatin isolated from starved and starved-refed cells (30 min after serum addition) and analysed as in Fig.1c to determine Pol-I binding to rDNA promoter (Prom), transcribed region (TrR) or non-transcribed region (nTrR). The value of each bar represents the difference between the signals from the specific antibody and from the negative control (an appropriate IgG) expressed as % from total chromatin input. Standard deviations from three independent experiments are shown; n=3.

B. ChIP assays were performed using antibodies specific to human histone H3 and chromatin isolated from starved and starved-refed cells (30 min after serum addition) and analysed as in Fig.1c to determine H3 occupancy at rDNA promoter (Prom), transcribed region (TrR) or non-transcribed region (nTrR). The value of each bar represents the difference between the signals from the specific antibody and from the negative control (an appropriate IgG) expressed as % from total chromatin input. Standard deviations from three independent experiments are shown; n=3.

C. Chromatin was isolated from starved and starved-refed cells (30 min after serum addition) and subjected for the first round of immunoprecipitation using antibody specific to Pol-I subunit A135. After elution chromatin was subjected to the second IP round using antibody specific to histone H3 and analysed by qPCR as in Fig 1c. The value of each bar represents the difference between the signals from the specific antibody and from the negative control (an appropriate IgG) expressed as % from total chromatin input. Standard deviations from three independent experiments are shown; n=3.

D. The raw data generated in the experiment described above (Supplementary Fig. 6c). The value of each bar represents the signal of either specific antibody (H3) or the negative control (IgG). Note that we observe a considerable (and unexplained) increase in the level of IgG signal in refed cells. Standard deviations from three independent experiments are shown; n=3.

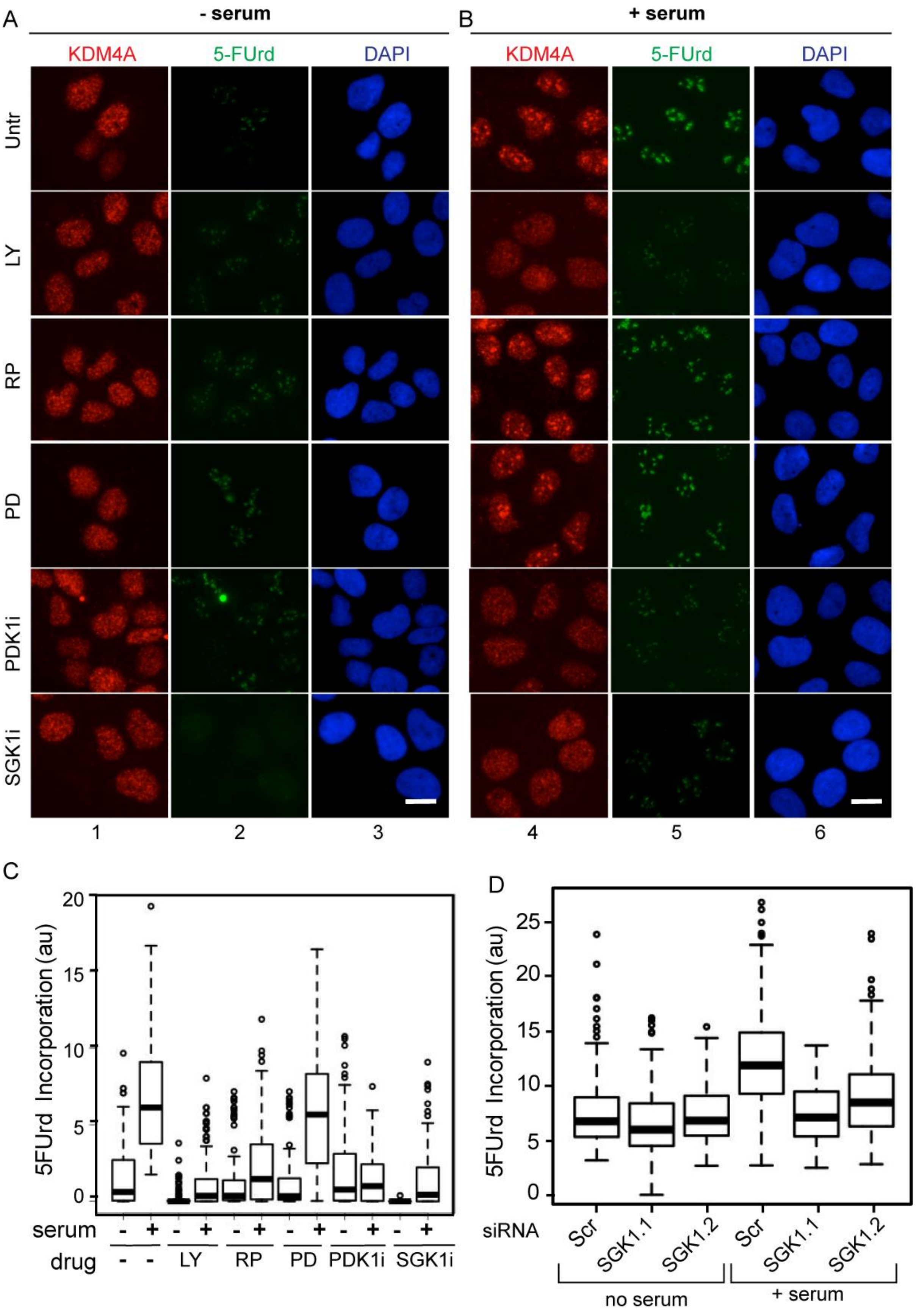

A
- serum
B
+ serum
KDM4A
5-FUrd
DAPI
Untr
LY
RP
PD
PDK1i
SGK1i
1
2
3
4
5
6
C
5FUrd Incorporation (au)
serum
drug
LY
RP
PD
PDK1i
SGK1i
D
siRNA
Scr
SGK1.1
SGK1.2
no serum
+ serum

Supplementary Figure 7. Effects of the various inhibitors in serum starved cells.
A-B. U2OS cells were serum starved for 24 hours and either refed with serum (**B**) or left starved (**A**) in the presence of 5-FUrd (same incubation time for starved and refed cells) and PI3K inhibitor LY294002 (LY), mTOR inhibitor rapamycin (RP), MAPK inhibitor PD98059 (PD), PDK1 inhibitor or SGK1 inhibitor, as indicated. Control cells were left untreated (Untr). 30 min after serum addition cells were fixed and analysed by indirect immunofluorescence using antibodies specific to human KDM4A (Ab1 Bethyl Lab, panels 1) and 5-FUrd (panels 2); nuclear DNA was stained by DAPI (panels 3). Bar = 5 μM.
C. Quantification of 5-FUrd staining in shown in panels A and B done as in Fig. 2e. The differences between serum-refeed cells treated with DMSO and all other populations are statistically significant (p-value < 10^{-32} for all populations except the PD-treated samples for which the p-value is 0.01, Wilcoxon test); n=3.
D. U2OS cells were transfected by the indicated siRNAs in Figure 5C, and either refed with serum or left starved in the presence of 5-FUrd. 5FUrd staining was quantified in the cell populations as described Fig. 2F. The differences between the cell population transfected with both SGK1 siRNAs and Scr siRNA in serum refeed cells are statistically significant (p-value < 10^{-12}, Wilcoxon test). The median values are shown as horizontal lines. Outliers are shown as open circles; n=3.

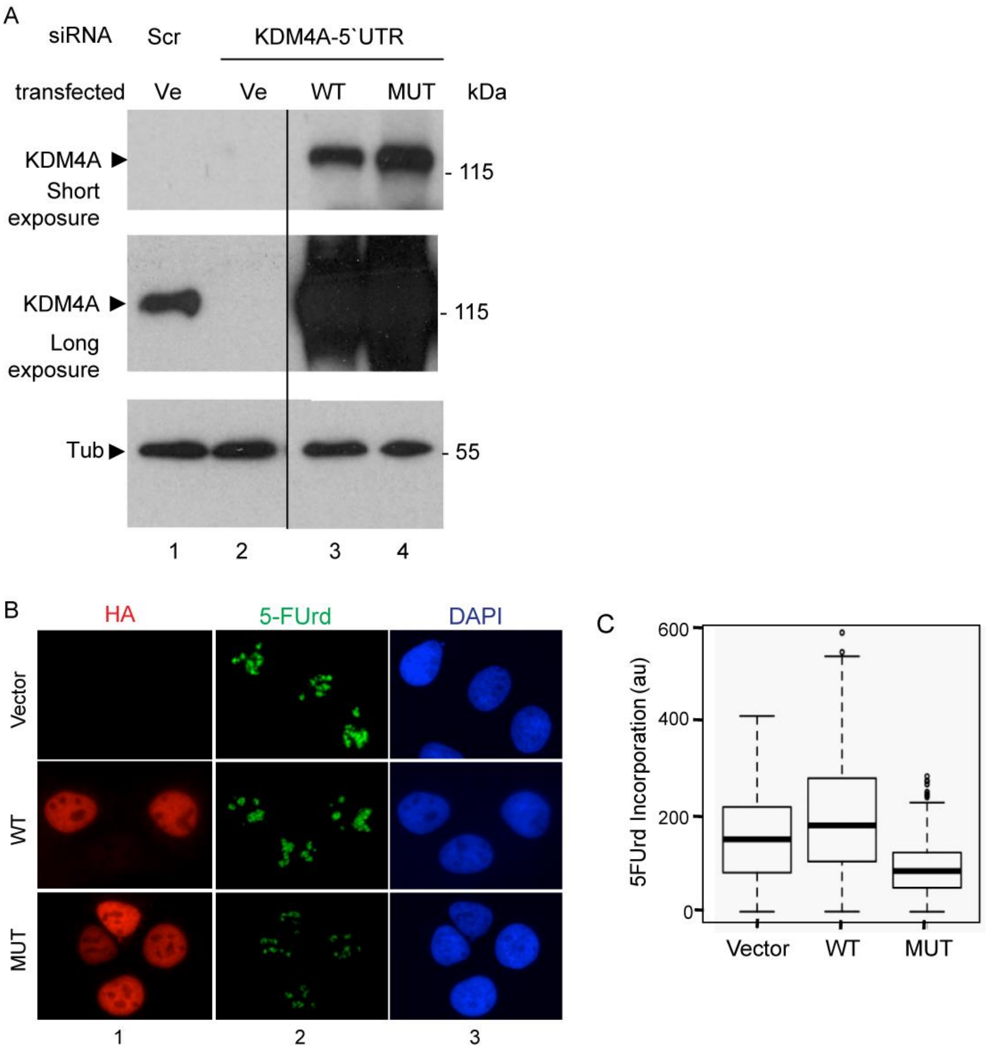

Supplementary Figure 8. Catalytically inactive KDM4A mutant functions as a dominant negative mutant.

A. U2OS cells were transfected by the indicated siRNAs and expression vectors for KDM4A wild type or mutant (or empty pcDNA3 vector, first two samples), and treated as in Fig. 5. Whole cell extracts were then analysed by Western blot using antibodies specific to KDM4A and Tubulin as a loading control. Two different exposures of the KDM4A Western blot are shown. The vertical lane indicates the localisation of a cut during figure preparation. Positions of molecular weight markers (PageRuller, Thermo Scientific) are indicated.

B. U2OS cells were electroporated with an expression vector encoding HA-tagged KDM4A either wild type (wt) or catalytically inactive mutant (mut) or with empty vector (Vector). Cells were grown for 24 hours and then starved for another 24 hours before serum refeeding in the presence of 5-FUrd. 30 min after serum addition cells were fixed and analysed by indirect immunofluorescence using antibodies specific to HA tag (panels 1), 5-FUrd (panels 2); nuclear DNA was stained by DAPI (panels 3).

C. Quantification of 5-FUrd staining from the experiment shown in panel A done as described in Fig. 2e.The differences between the cell population transfected with KDM4A-wt and KDM4A-mut in serum refeed cells are statistically significant (p-value < 10^{-13}, Wilcoxon test). The median values are shown as horizontal lines. Outliers are shown as open circles; n=3.n=3.

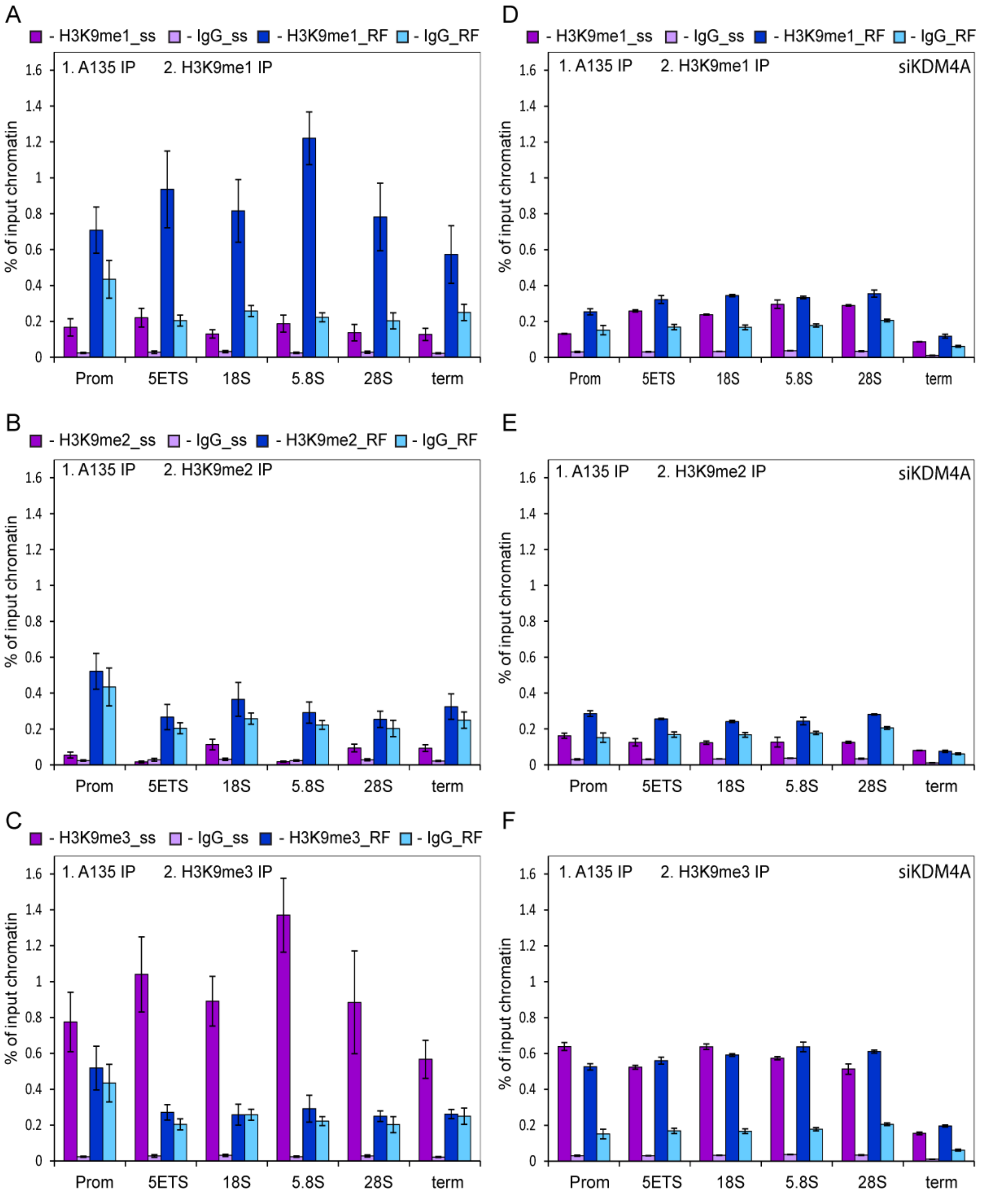

Supplementary Figure 9. Raw data of sChIPs shown in Fig. 6b-e

A-C. ChIP assay were performed as described in Fig. 6b and c. The value of each bar represents the signal either from the specific antibody or from the negative control (an appropriate IgG) expressed as % from total chromatin input. Signals for specific regions of the transcribed region (5'ETS, 18S, 5.8S and 28S) are shown. The signals are not normalized to the

specific H3 signal. Note that we observe a considerable (and unexplained) increase in the level of IgG signal in refed cells. Standard deviations from three independent experiments are shown; n=3.

D-F. ChIP assay were performed as described in Fig. 6d and e. The value of each bar represents the signal either from the specific antibody or from the negative control (an appropriate IgG) expressed as % from total chromatin input. Signals for specific regions of the transcribed region (5'ETS, 18S, 5.8S and 28S) are shown. The signals are not normalized to the specific H3 signal. Note that we observe a considerable (and unexplained) increase in the level of IgG signal in refed cells. Standard deviations from three independent experiments are shown; n=3.

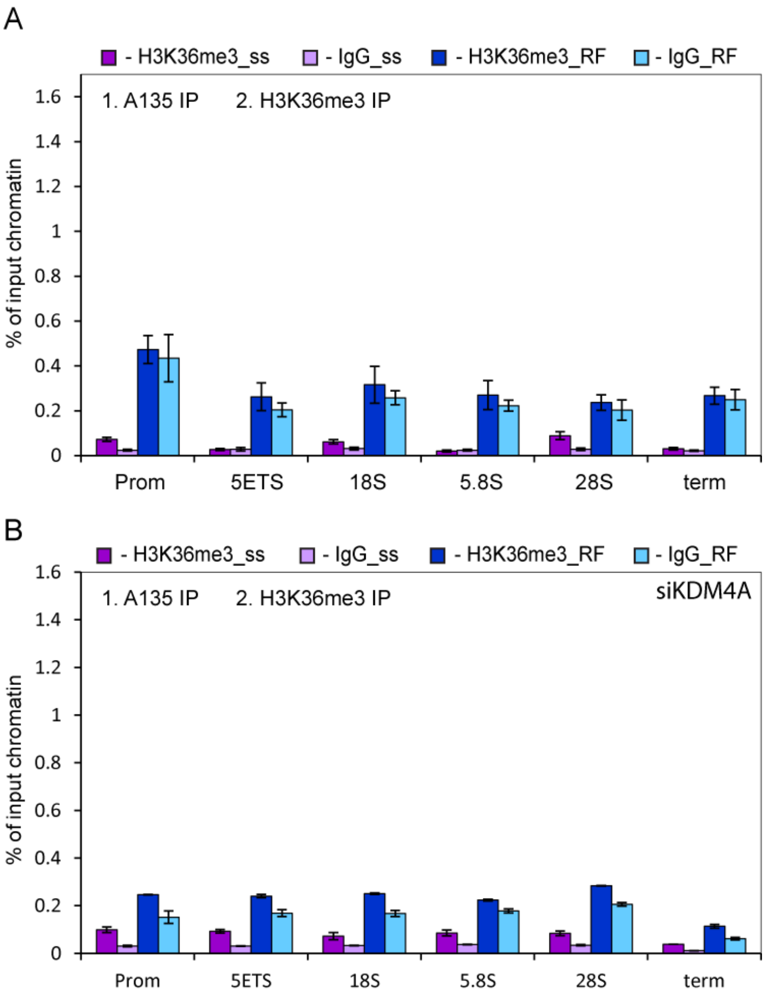

Supplementary Figure 10. No detectable enrichment in H3K36me3 at euchromatic rDNA.
A. ChIP assays were performed as in Fig. 6b-c. The value of each bar represents the signal either from the specific antibody or from the negative control (an appropriate IgG) expressed as % from total chromatin input. Signals for specific regions of the transcribed region (5'ETS, 18S, 5.8S and 28S) are shown. The signals are not normalized to the specific H3 signal. Note that we observe a considerable (and unexplained) increase in the level of IgG signal in refed cells. Standard deviations from three independent experiments are shown; n=3.
B. ChIP assays were performed as in Fig. 6d-e. The value of each bar represents the signal either from the specific antibody or from the negative control (an appropriate IgG) expressed as % from total chromatin input. Signals for specific regions of the transcribed region (5'ETS, 18S, 5.8S and 28S) are shown. The signals are not normalized to the specific H3 signal. Note that we observe a considerable (and unexplained) increase in the level of IgG signal in refed cells. Standard deviations from three independent experiments are shown; n=3.

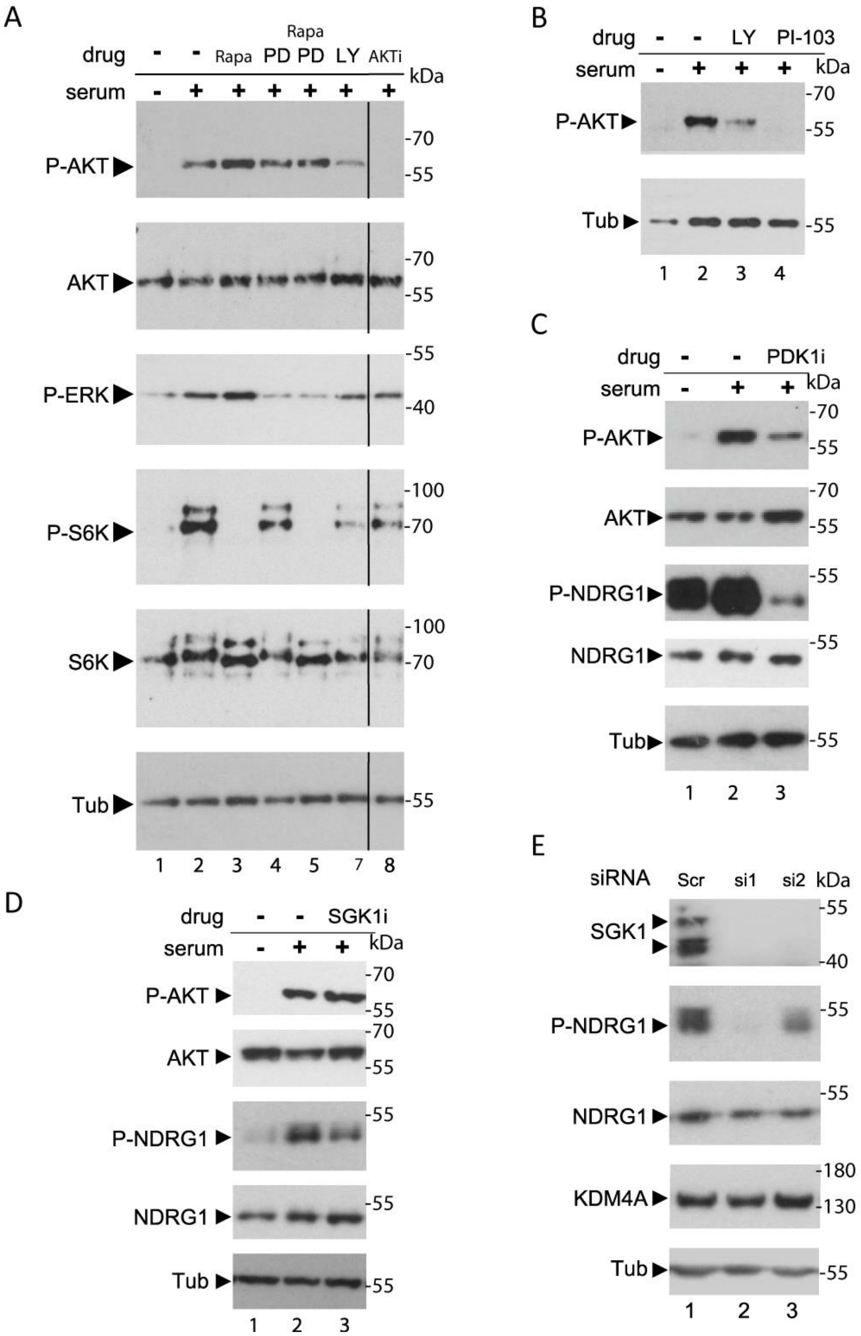

Supplementary Figure 11. Efficiency of kinase inhibition.

A-D. U2OS cells were treated as in Fig. 7 and Fig. 8. Whole cell extracts were then analyzed by Western blot using antibodies specific to the indicated proteins : phosphorylated AKT (Ser 473), total AKT, phosphorylated ERK1/2, phosphorylated S6K (Thr389), total S6K, phosphorylated NDRG1, total NRDG1 and Tubulin as a loading control, as indicated. The vertical lane in A indicates the localisation of a cut during figure preparation.

E. U2OS cells were transfected with the indicated siRNAs and treated as in Fig. 8. Whole cell extracts were then analyzed by Western blot using antibodies specific to the indicated proteins: SGK1, phosphorylated AKT (Ser 473), total AKT, phosphorylated NDRG1, total NRDG1, KDM4A and Tubulin as a loading control, as indicated.

Positions of molecular weight markers (PageRuller, Thermo Scientific) are indicated.

Supplementary Tables

Table S1. List of antibodies used in ChIP assay.

Cells per IP	Antigen	Source	Cat. number	Amount per IP
$1x10^6$	hA135 (N-17)	Santa-Cruz	Sc-17913	5 µg
	KDM4A/JMJD2A	Bethyl	A300-861A	5 µg
	KDM4A/JMJD2A	Sigma	SAB3500095	5 µg
	H3	Abcam	1791	5 µg
	H3K9me1	Diagenode	pAb-065-050	4 µg
	H3K9me2	Diagenode	pAb-060-050	4 µg
	H3K9me3	Diagenode	MAb-146-050	4 µg
	H3K36me3	Diagenode	CS-058-100	20 µl
	rabbit IgG	Sigma	I5006	5 µg
	sheep IgG	Sigma	I5131	5 µg
	mouse IgG	Sigma	I5381	5 µg

Table S2. List of primers and probes used in ChIP assay.

Tetraplex	Region	Name	Position*	Sequence (5'-3')
I	Promoter	Pr_F	42941-42956	GCCCCGGGGGAGGTAT
		Pr_R	42995-17	GAGGACAGCGTGTCAGCAATAA
		Pr_p	42958-42982	FAM-TCTTTCGCTCCGAGTCGGCATTTTG-BHQ2
	18S	18S_F	4390-4404	CCCGCCCCTTGCCTC
		18S_R	4426-4443	CCGCGGGACACTCAGCTA
		18S_p	4406-4424	RED-CGGCGCCCCCTCGATGCTC-BHQ2
	5.8S	5.8_F	6452-6470	TGTGAAACCTTCCGACCCC
		5.8_R	6509-6524	GGGGTTGCGTCAGGCC
		5.8_p	6475-6503	Cy5-CGGAGTCCGGTCCCGTTTGCTGTCTCGTC-BHQ2
	IGS2	I2_F	35996-36012	CGGGCCTTGGCAGATTC
		I2_R	36107-36126	CGCGCGTAGAGGAGAGATTT
		I2_p	36067-36089	HEX-CCCCGAGTGACTGTGGCTCGCAC-BHQ2
II	5`ETS	5E_F	1783-1799	CCGTGCCGAGTCGTGAC
		5E_F	1844-1859	CGCTTTCCCAGGGCCA
		5E_p	1803-1821	Cy5-TGCCGACGACCGCGTTTGC-BHQ2
	28S	28S_F	11740-11763	GAGATTCCCACTGTCCCTACCTAC
		28S_R	11792-11808	CTGATTCCGCCAAGCCC
		28S_p	11765-11788	FAM-ATCCAGCGAAACCACAGCCAAGGG-BHQ2
	IGS1	I1_F	20171-20187	GCGAGGCACAGCGCATC
		I1_R	20252-20271	GGCATCAGCTCCAACTCCAG
		I1_p	20189-20211	RED-CTTTGGAAGCCGCGGCAACGCCT-BHQ2

*-positions correspond to numeration in human rDNA complete repeating unit, accession number: U13369
BHQ2 - BlackHoleQuencher2
FAM - 6-carboxyfluorescein
HEX - Hexachloro-fluorescein
RED – Texas Red

Table S3. List of siRNA (sequence of the plus strand).

KDM4A-1	GUUCGAGAGUUCCGCAAGAdTdT
KDM4A-2	GUAUGAUCUUCCAGACUUAdTdT
KDM4A-3	GGGAUUCUAUCUCUUCUGAdTdT
KDM4A-5'UTR	CUGACUAAAGGGACCUCAAdTdT
Non-targeting (siCtle)	CAUGUCAUGUGUCACAUCUdTdT
SGK1-1	CAGCUGAAAUGUACGACAAdTdT
SGK1-2	GGCUACCUGCAUUCACUGAdTdT

Table S4. List of primers used in RT-PCR and ChIP*.

KDM4A	Forward<br>Reverse	CACATCCACCGGACTTCTTT<br>GTTCTCGGTGTTCAGCCAAT
KDM4B	Forward<br>Reverse	TCAGGGTTGTACCCGAAGAG<br>GCCCACCTTTGCAGAATAAA
KDM4C	Forward<br>Reverse	GCTTGCGAGAAGGTCATTTC<br>AGACAGTCTCGGCTCACGAT
rRNA	Forward	GCCCGGGAGAGCACGAC
	Reverse	GCGTGTCAGGCGTTCTCG
UBF	Forward	CCCCTTATTTCCGCTTCTTC
	Reverse	TTTCGCTCGAACTCCTGTTT
RPA194	Forward<br>Reverse	ACATTTGGATTCGCCTTCAG<br>TGTCTGGGCAGATGACTGAG
P0*	Foward<br>Reverse	GGCGACCTGGAAGTCCAACT<br>CCATCAGCACCACAGCCTTC
CDC6P*	Forward<br>Reverse	CAGTTTGTTCAGGGGCTTGT<br>GCTCAGCTCTTTTCCCTTCA
GAPDHP*	Forward<br>Reverse	CCAATGTGTCCGTCGTGGATCT<br>GTTGAAGTCGCAGGAGACAACC
